# Host interneurons mediate plasticity reactivated by embryonic inhibitory cell transplantation in mouse visual cortex

**DOI:** 10.1038/s41467-021-21097-4

**Published:** 2021-02-08

**Authors:** XiaoTing Zheng, Kirstie J. Salinas, Dario X. Figueroa Velez, Taylor Nakayama, Xiaoxiao Lin, Dhruba Banerjee, Xiangmin Xu, Sunil P. Gandhi

**Affiliations:** 1 Department of Neurobiology and Behavior, University of California, Irvine, Irvine, CA 92697-2715 USA; 2 Department of Anatomy and Neurobiology, University of California, Irvine, Irvine, CA 92697-2715 USA; 3 Department of Biomedical Engineering, University of California, Irvine, Irvine, CA 92697-2715 USA; 4 Department of Microbiology and Molecular Genetics, University of California, Irvine, Irvine, CA 92697-2715 USA; 5 Center for the Neurobiology of Learning and Memory, University of California, Irvine, Irvine, CA 92697-2715 USA

**Keywords:** Neuronal development, Neuronal physiology, Inhibition, Regeneration and repair in the nervous system, Visual system

## Abstract

The adult brain lacks sensitivity to changes in the sensory environment found in the juvenile brain. The transplantation of embryonic interneurons has been shown to restore juvenile plasticity to the adult host visual cortex. It is unclear whether transplanted interneurons directly mediate the renewed cortical plasticity or whether these cells act indirectly by modifying the host interneuron circuitry. Here we find that the transplant-induced reorganization of mouse host circuits is specifically mediated by Neuregulin (NRG1)/ErbB4 signaling in host parvalbumin (PV) interneurons. Brief visual deprivation reduces the visual activity of host PV interneurons but has negligible effects on the responses of transplanted PV interneurons. Exogenous NRG1 both prevents the deprivation-induced reduction in the visual responses of host PV interneurons and blocks the transplant-induced reorganization of the host circuit. While deletion of ErbB4 receptors from host PV interneurons blocks cortical plasticity in the transplant recipients, deletion of the receptors from the donor PV interneurons does not. Altogether, our results indicate that transplanted embryonic interneurons reactivate cortical plasticity by rejuvenating the function of host PV interneurons.

## Abstract (web summary)

Transplantation of embryonic interneurons can restore juvenile plasticity to the adult host visual cortex. Here, the authors show that transplanted embryonic interneurons reactivate cortical plasticity via Neuregulin/ErbB4 signaling in host parvalbumin interneurons.

## Introduction

The modification of inhibitory signaling in the adult brain using the transplantation of embryonic inhibitory neurons has emerged as a promising strategy to recondition dysfunctional or diseased neural circuits^1–4^. The mechanism underlying transplant-induced cortical reorganization is not well understood. Previously, we demonstrated that embryonic interneuron transplantation reverses visual deficits in adult mice by reactivating a second critical period for cortical plasticity^5^. As the reactivated window for plasticity coincides with the developmental critical period of the donor animal, it has been hypothesized that transplanted cells add a juvenile-like inhibitory circuit to the mature brain that executes a cell-intrinsically timed developmental program for cortical plasticity^5–8^.

During the development of the visual cortex, the experience-dependent plasticity of excitatory circuits is gated by neuregulin 1 (NRG1)/ErbB4-dependent changes within parvalbumin (PV) GABAergic interneurons^9–11^. NRG1 regulates the excitability of PV interneurons by binding to ErbB4 receptors on PV interneurons^12–14^. Upon brief monocular deprivation (MD) by eyelid suture, NRG1/ErbB4 signaling mediates a rapid loss of excitatory drive onto PV interneurons. The resulting reduction in PV cell activity is necessary for the subsequent reorganization of the excitatory circuit^10,11^. The level of NRG1 within PV interneurons peaks during the early juvenile development but gradually decreases as the cortex matures^11,15^. These results suggest that NRG1/ErbB4 signaling may be involved in juvenile cortical plasticity.

Given our hypothesis that transplanted cells induce cortical plasticity by executing a developmental program similar to that observed in normal development, we predicted that MD would rapidly reduce transplant PV cell activity. Surprisingly, we find that brief MD greatly reduces the visual activity of adult host PV interneurons but has little effect on transplanted interneurons. Moreover, we show that NRG1/ErbB4 signaling is essential for transplant-reactivated cortical plasticity, and it depends on the ErbB4 receptor activity in the host but not the transplanted PV interneurons. These results suggest that interneuron transplantation reactivates NRG1/ErbB4, a signaling pathway normally involved in developmental cortical plasticity, within the adult host PV interneurons and enables a second critical period.

## Results

### Sensory deprivation reduces the responses of juvenile PV interneurons

**Fig. 1:**
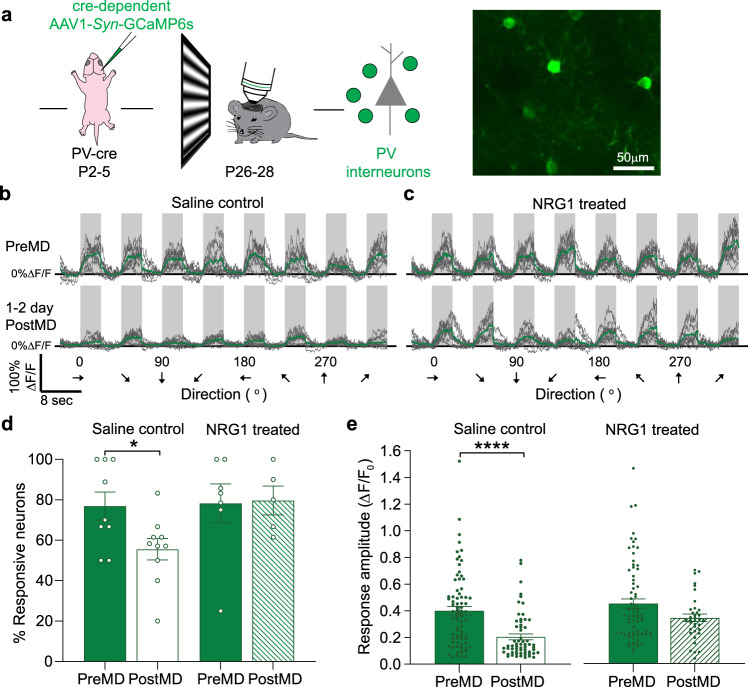
Sensitivity of juvenile PV interneurons to brief MD depends on NRG1 signaling. **a** Left: schematic of experimental design for viral labeling PV interneurons in neonates at postnatal days (P) 2–5 and Ca^2+^ imaging in critical period animals at P26-28. Right: in vivo image of visually responsive PV interneurons (green). Scale bar = 50 µm. This experiment was performed independently in eight animals total (6 saline group, 2 NRG1 group). **b** Visually evoked responses of PV interneurons through the deprived eye before and after monocular deprivation (MD) in saline controls. **c** Visually evoked responses of PV interneurons through the deprived eye before and after MD in neuregulin 1 (NRG1) treated mice. **d** 1D MD significantly decreased the percent of responsive PV interneurons (PreMD: *n* = 9 fields from 3 mice, solid green; PostMD: *n* = 10 fields from 3 mice, open green; *p = 0.027, *t*-test with Welch’s correction, two-tailed). Exogenous NRG1 prevented the reduction in PV cell responsiveness (PreMD: *n* = 7 fields from 2 mice, solid green; PostMD: *n* = 5 fields from 2 mice, dashed green; *p* = 0.876, Mann–Whitney *U*-test, two-tailed). **e** 1D MD also reduced the response amplitude of PV interneurons through the contralateral, deprived eye stimulation (Δ*F*/*F*_0_: PreMD, 0.401 ± 0.031, *n* = 78 cells from 5 mice, solid green; PostMD, 0.205 ± 0.022, *n* = 61 cells from 3 mice, open green; ****p = 0.00000046, Mann–Whitney *U*-test, two-tailed). Exogenous NRG1 blocked the reduction in response amplitude of PV interneurons. (Δ*F*/*F*_0_: PreMD, 0.449 ± 0.035, *n* = 73 cells from 3 mice, solid green; PostMD, 0.342 ± 0.028, *n* = 36 cells from 2 mice, dashed green; *p* = 0.266, Mann–Whitney *U*-test, two-tailed). Data in **d** and **e** are mean ± SEM.

Previous studies have shown that visual deprivation rapidly decreases the responses of PV interneurons, and a series of in vitro experiments shows that the reduction can be prevented by treating PV interneurons with exogenous NRG1^11^. Here, we confirmed previous results in juvenile critical period (P26-28) mice using in vivo two-photon calcium (Ca^2+^) imaging of GCaMP6s. We recorded visually evoked activity of layer 2/3 PV cells in binocular visual cortex using cre-dependent AAV1-Syn-GCaMP6s injections into PV-cre mice (Fig. 1a–c). One to 2 days of MD rapidly reduced the overall responsiveness of PV interneurons. We determined the overall responsiveness by calculating: (a) the percentage of responsive cells and (b) response strength of responsive cells. We found that one day MD reduced both the percentage of responsive PV interneurons (Fig. 1d) and response amplitude (Fig. 1b, e) of PV interneurons, consistent with previous studies^10,11^. Moreover, we showed that treatment with exogenous NRG1 during MD prevented the reduction in PV cell activity (Fig. 1c, d, e).

### Transplanted PV interneurons differentiate and synaptically integrate into the host circuit

With the goal of examining how visual experience differentially affects the transplant and host PV interneurons, we first transplanted embryonic GABAergic progenitors from medial ganglionic eminence (MGE) into the visual cortex of adult mice. We confirmed that the transplanted PV interneurons dispersed and expressed mature interneuron markers at the time of reactivated critical period at 35 days after transplantation (DAT). We found transplanted tdTomato+ cells in all layers of the visual cortex (Supplementary Fig. 1a). Immunostaining revealed that >80% of these transplanted cells were PV+ (Supplementary Fig. 1c–e, g).

**Fig. 2:**
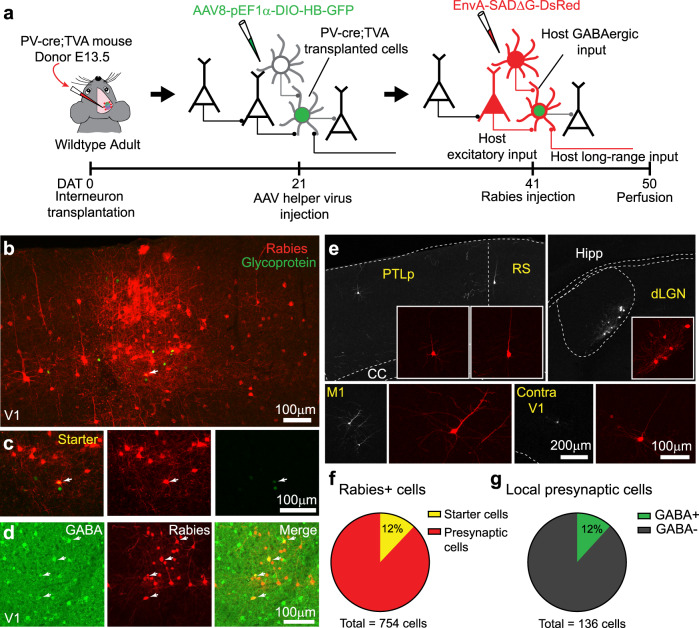
Transplanted PV interneurons are integrated into the host circuit. **a** Experimental design. **b**, **c**, **e** Rabies tracing experiments were successfully performed in four transplant recipients. The results were qualitatively similar. The transplanted cells received both local and long-range connections. **b** Identification of transplanted starter cells by co-expression of rabies glycoprotein (green) and rabies virus (red). Scale bar = 100 µm, 50 µm max projection. **c** Magnified images of the starter cell in **b**. Scale bar = 100 µm, 3 µm max projection. **d** Transplanted PV cells received both GABAergic (yellow) and putative excitatory inputs (red) from the host cortex. Scale bar = 100 µm. Immunostaining of GABA was performed in sections taken from one mouse. Quantification shown in **f** and **g**. **e** Transplanted PV cells received long-range inputs from various brain regions. Scale bar = 200 µm, 100 µm. **f** Quantification of transplanted starter cells (total number of rabies+ cells counted: 754, number rabies+/GFP+ starter cells: 91, and number of rabies+ only cells: 663, 1 mouse). **g** Quantification of local presynaptic cells that provided inputs to starter transplanted cells (total number of rabies+ presynaptic cells: 136, number of GABA+/rabies+ cells: 16, number of GABA-/rabies+ cells: 120, 1 mouse).

To participate in cortical plasticity, transplanted cells must form proper synapses with the host circuit. Previous studies have performed in vitro electrophysiology in slice to show that transplanted cells form and receive connections from the host circuit^7,16,17^. However, electrophysiological recording can only reveal the synaptic connections within the local circuit. To identify both local and long-range inputs to transplanted PV interneurons, we performed retrograde tracing using rabies virus (Fig. 2a and “Methods”). We collected tissue from embryos that selectively express avian tumor receptor A (TVA) in PV interneurons and transplanted into the binocular visual cortex of wild-type adult mice. We then injected cre-dependent rabies helper virus 3 weeks after transplantation. The rabies virus was injected at 6–7 weeks after transplantation. The cre-LoxP-based approach restricted the helper virus and initial rabies virus infection to transplanted PV interneurons (see “Methods”)^18–20^. Out of all the rabies-labeled neurons in V1, 12% were transplanted starter cells that expressed both rabies virus and nuclear green fluorescent protein (GFP) (Fig. 2f). The transplanted PV starter cells received both local (Fig. 2b, c) and long-range inputs (Fig. 2e) from the host brain like PV interneurons in normal adult mice (Supplementary Fig. 2)^21^. The local connections included both GABAergic (12%) and putative excitatory inputs (Fig. 2d, f). Previous studies using the same rabies virus and approach demonstrate that with no rabies glycoprotein (RG) present, rabies virus only infects the TVA-expressing starter cells but not the presynaptic partners^19^. Altogether, these results suggest that the transplanted cells had differentiated into mature PV interneurons and received proper synaptic inputs from host brain at the time of reactivated critical period for plasticity.

### Sensory deprivation does not alter the responses of transplanted PV interneurons

**Fig. 3:**
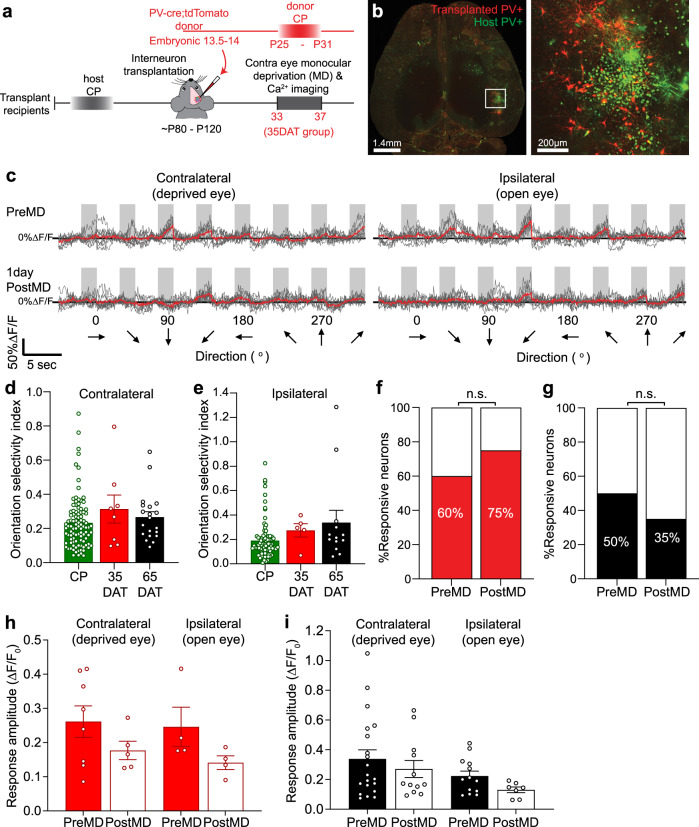
Transplanted PV interneurons are not sensitive to MD. **a** Experimental timeline. The calcium imaging of transplanted PV interneurons was performed at 33–35 days after transplant (35DAT) or 65–70DAT. **b** Left: whole brain image of transplant recipient cleared using iDISCO+ method, scale bar = 1.4 mm. Right: a magnified view of the outlined region in the left panel. Transplanted PV interneurons are in red, and AAV1-GCaMP6s-labeled host PV interneurons are in green and transplanted PV interneurons are in yellow, scale bar = 200 µm. This experiment was performed in one transplant recipient. **c** Visually evoked responses of an example transplanted PV interneuron. **d**, **e** There was no difference in the orientation selectivity index between critical period (CP, green) PV interneurons, and 35DAT (red) and 65DAT (black) transplanted PV interneurons. **d** Orientation selectivity index for the contralateral responses (CP: *n* = 98 cells from 3 mice; 35DAT: *n* = 8 cells from 6 mice; 65DAT: *n* = 20 cells from 5 mice; *p* = 0.339, Kruskal–Wallis ANOVA test). **e** Orientation selectivity index for the ipsilateral responses (CP: *n* = 84 cells from 3 mice; 35DAT: *n* = 5 cells from 5 mice; 65DAT: *n* = 13 cells from 5 mice; CP vs. 35DAT, *p* = 0.144; 65DAT vs. 35DAT, *p* = 0.999 Kruskal–Wallis ANOVA with Dunn’s multiple comparison test). **f** Responsiveness of transplanted PV interneurons at 35DAT remained unchanged after 1D MD (PreMD: *n* = 15 cells from 6 mice; PostMD: *n* = 8 cells from 3 mice, *p* = 0.657, Fisher’s exact test). **g** The percentage of responsive transplanted PV interneurons also remained unchanged after MD for the 65DAT group (PreMD: *n* = 50 cells from 5 mice; PostMD: *n* = 37 cells, *p* = 0.194, Fisher’s exact test). **h** The response amplitude of transplanted PV interneurons did not change after 1D MD for either the contralateral or ipsilateral eye (Contra PreMD: *n* = 8 cells from 6 mice, solid red; PostMD: *n* = 5 cells from 4 mice, open red; *p* = 0.284, Mann–Whitney *U*-test, two-tailed; Ipsi PreMD and PostMD: *n* = 4 cells from 4 mice; *p* = 0.114, Mann–Whitney *U*-test, two-tailed). **i** MD also did not significantly alter the response amplitude of transplanted PV interneurons at 65DAT through the contralateral eye (PreMD: *n* = 20 cells from 5 mice, solid black; PostMD: *n* = 12 cells from 5 mice, open black; *p* = 0.604, Mann–Whitney test, two-tailed) or ipsilateral eye stimulation (PreMD: *n* = 13 cells from 5 mice, solid black; PostMD: *n* = 7 cells from 2 mice, open black; *p* = 0.114, Mann–Whitney test, two-tailed). Data in **d**, **e**, **h**, and **i** are represented as mean ± SEM.

To test whether transplanted cells were responsive to sensory manipulation, we next performed in vivo time-lapse two-photon Ca^2+^ imaging to examine the effects of brief MD on layer 2/3 transplanted PV cell activity (Fig. 3). First, we labeled the transplanted PV cells with either cre-dependent or synapsin-driven AAV1 GCaMP6s (Fig. 3a). The Ca^2+^ imaging was performed at 33–35DAT when the age of transplanted PV interneurons was equivalent to P26-P28 critical period PV cells. We first compared the visual tuning properties of the 35DAT transplanted PV cells to that of the critical period PV cells and 65DAT transplanted cells. Although the 35DAT transplanted PV interneurons had slightly higher orientation selectivity index, there was no significant difference between the three groups for both contralateral (Fig. 3d) and ipsilateral eyes (Fig. 3e). After the first day of imaging, the eye contralateral to the imaged cortex was closed by eyelid suture for 20–24 h. Whereas MD reduced the responsiveness and response amplitude of PV cell activity in juvenile critical period mice (Fig. 1), we were surprised to find no change in the overall responsiveness (Fig. 3f) or the response amplitude of transplanted PV interneurons (Fig. 3h). MD also showed no effect on the overall responsiveness or response amplitude of 65DAT transplanted PV interneurons, at the time when they had passed their critical period.

### Inactivation of transplanted PV interneurons does not prevent OD plasticity

To further investigate whether the activity of PV interneurons was necessary for transplant-induced cortical plasticity, we suppressed the activity of the transplanted interneurons during MD and measured ocular dominance plasticity (ODP) of the recipients. We crossed PV-cre breeders with floxed-DREADD mice (JAX, 026219) to genetically express DREADD receptors in PV interneuron progenitors. A previous study has demonstrated the viability of this line transgenic line for transplantation and in vivo study^22^. We harvested MGE cells from E13.5 PV-cre;DREADD embryos and transplanted them into wild-type adult visual cortex. We performed intrinsic signal optical imaging (ISOI) to measure OD index before and after 4 days of MD. Two doses of Clozapine-N-oxide (CNO; 5 mg/kg) were given at 10–12 h intervals for 4 days during deprivation (Supplementary Fig. 3a). It has been shown that CNO can reduce the visual responses of DREADD-expressing PV interneurons within 1 h of injection, and the effect can last for up to 12 h^10^. Inactivation of transplanted cells did not prevent ODP in the adult transplant recipients (Supplementary Fig. 3b, c). Consistent with a previous study^23^, once integrated into the host circuit, the transplanted interneurons may not directly contribute to the expression of ODP of the recipients.

### Sensory deprivation reduces the responsiveness of host PV interneurons

**Fig. 4:**
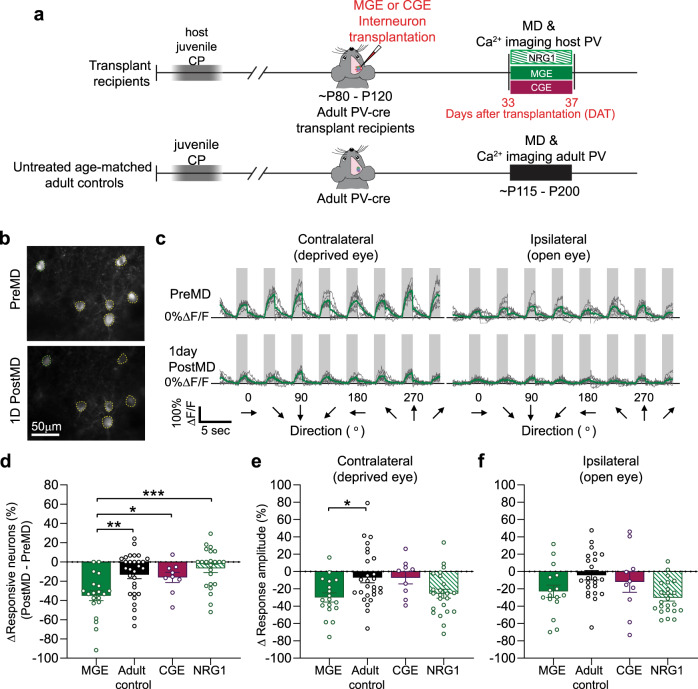
Transplant-induced sensitivity of host PV interneurons to brief MD depends upon NRG1 signaling. **a** Experimental timeline. Top: calcium imaging of host PV interneurons in MGE transplant recipients (green, MGE), MGE recipients treated with NRG1 (dashed green, NRG1) or CGE transplant recipients (magenta, CGE) were performed at 33–35 days after transplantation. Bottom: imaging of PV interneurons in untreated adult control mice was performed around postnatal day (P) 115 (black, adult control). **b** In vivo image of GCaMP6s-labeled host PV interneurons in a MGE transplant recipient. Responsive cells are outlined in yellow and green. Scale bar = 50 µm. In vivo Ca^2+^ imaging of PV interneurons were performed independently in 25 mice (17 transplant recipients and 8 wild-type mice). **c** Visually evoked responses at the peak spatial frequency for the cell outlined in green in **b**. **d** 1D MD reduced the overall responsiveness of host PV interneurons in 35DAT transplant recipients (MGE: *n* = 20 fields from 6 mice; adult control: *n* = 30 fields from 8 mice; 35DAT CGE: *n* = 9 fields from 5 mice; NRG1: *n* = 23 fields from 6 mice; 35DAT MGE vs. adult control: **p = 0.0054; 35DAT MGE vs. 35DAT CGE: *p = 0.048; 35DAT MGE vs. NRG1: ***p = 0.0004, Brown–Forsythe ANOVA followed by Dunnett’s T3 multiple comparisons test). **e**, **f** 1D MD resulted in a large reduction in the response amplitude of 35DAT host PV interneurons through both the deprived (**e**) and open eyes (**f**). However, exogenous NRG1 did not prevent the reduction in the response amplitude of host PV interneurons (Contra: 35DAT MGE, *n* = 18 fields from 6 mice; adult control, *n* = 27 fields from 8 mice; CGE, *n* = 9 fields from 5 mice; NRG1, *n* = 23 fields from 6 mice; 35DAT MGE vs. adult control, *p = 0.019; 35DAT MGE vs. 35DAT CGE: *p* = 0.056; 35DAT MGE vs. NRG1: *p* = 0.914; Ipsi: 35DAT MGE, *n* = 16 fields from 6 mice; adult control, *n* = 23 fields from 8 mice; CGE, *n* = 10 fields from 5 mice; NRG1, *n* = 23 fields from 8 mice; 35DAT MGE vs. adult control, *p* = 0.125; 35DAT MGE vs. 35DAT CGE: *p* = 0.819; 35DAT MGE vs. NRG1: *p* = 0.746, Brown–Forsythe and Welch ANOVA followed by Dunnett’s T3 multiple comparisons test). Data in **d**, **e**, and **f** are mean ± SEM.

**Fig. 5:**
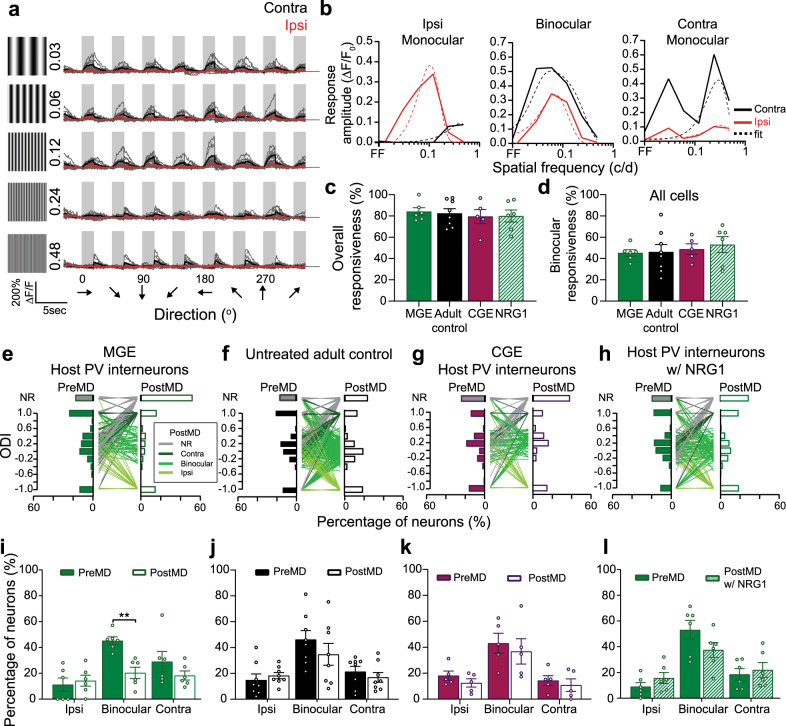
NRG1 prevents the selective reduction of binocular responses in host PV interneurons. **a**, Example binocular responses from a host PV interneuron at 35DAT before MD. Gray boxes represent when the visual gratings were shown. Gray traces represent individual trials. Black represents averaged responses for contralateral eye stimulation. Red represents averaged responses for ipsilateral eye stimulation. The peak spatial frequency for the contralateral responses is 0.12 c/d and 0.06 c/d for the ipsilateral responses in the example shown. **b** Examples of different types of spatial frequency responses: binocular, contralateral monocular, and ipsilateral monocular cells. The average responses (solid line) at each spatial frequency are overlaid with a difference of Gaussians fit (dashed line). **c** No difference found in the overall responsiveness between all four experimental groups (MGE: *n* = 6 mice, 84.14% ± 3.63, solid green; Adult control: *n* = 8 mice, 82.51% ± 4.33, black; CGE: *n* = 5 mice, 79.48% ± 6.57, magenta; NRG1: *n* = 6 mice, 79.99% ± 5.68, dashed green; *p* = 0.910, one-way ANOVA) before MD. **d** No difference found in the percentage of responsive binocular responses (MGE: *n* = 6 mice, 45.23% ± 3.00; adult control: *n* = 8 mice, 46.22% ± 6.99; CGE: *n* = 5 mice, 48.87% ± 5.00; NRG1: *n* = 6 mice, 53.11% ± 7.47; *p* = 0.816, one-way ANOVA). **e**–**h** Single-cell ODI distribution of PV interneurons before and after MD. The colors of the lines are determined by the cell’s PostMD ODI. Non-responsive cells (NR): gray; contralateral only cells: dark green; binocular cells: medium green; ipsilateral cells: light green. **e** 35DAT host PV interneurons with MGE transplant (*n* = 260 cells from 6 mice). **f** Untreated adult PV interneurons (*n* = 435 cells from 8 mice). **g** 35DAT host PV interneurons with CGE transplant (*n* = 150 cells from 5 mice). **h** 35DAT host PV interneurons treated with NRG1 (*n* = 248 cells from 6 mice). **i**–**l** Same data as **e**–**h** but plotted by animal. **i** 1D MD selectively reduced the percentage of responsive binocular PV interneurons (*n* = 6 mice, **p = 0.0018, two-way ANOVA followed by Bonferroni’s multiple comparison test). **j** No significant reduction in the percentage of responsive binocular PV interneurons after 1D MD in untreated, age-matched adult control (*n* = 8 mice, two-way ANOVA followed by Bonferroni’s multiple comparison test). **k** No significant reduction in the binocular host PV interneurons in adults with CGE transplant (*n* = 5 mice, two-way ANOVA followed by Bonferroni’s multiple comparison test). **l** NRG1 treatment prevented the reduction in responsiveness of binocular PV interneurons after MD (*n* = 6 mice, two-way ANOVA followed by Bonferroni’s multiple comparison test). Data in **c**, **d**, **i**, **j**, **k**, and **l** are mean ± SEM.

As transplanted cells failed to respond significantly to sensory deprivation and the inactivation of these cells did not prevent ODP, our results are inconsistent with the notion that transplanted cells recapitulate the developmental program for cortical reorganization. Next, we examined whether interneuron transplantation restored sensitivity to visual deprivation in host PV interneurons. To record the visually evoked responses of layer 2/3 host PV interneurons, we transplanted PV-tdTomato+ interneurons into PV-cre adult mice. Then, we labeled the host PV cells with cre-dependent synapsin-driven AAV1 GCaMP6s (Fig. 4a). Post-hoc immunostaining of virus-labeled cells confirmed that the majority of these host cells were PV-positive (>90%; Supplementary Fig. 4). We observed no difference in the baseline responsiveness of PV interneurons between experimental groups before MD (Fig. 5c). The tuning properties of PV interneurons were also not altered by transplantation (Supplementary Fig. 5a–c). We found no significant difference in median orientation selectivity index (Supplementary Fig. 5b) or peak spatial frequency (Supplementary Fig. 5c) in PV interneurons between MGE transplant recipients and adult controls before MD.

In contrast to the negligible effect we found in the transplant PV interneurons, the overall responsiveness of host PV interneurons was greatly reduced after 1 day of MD. We noticed that the decrease in the percentage (35–40%) of responsive PV interneurons (Fig. 4d, green) and the reduction of response amplitude (30%; Fig. 4e, green) were significantly greater in 35DAT MGE transplant recipients compared to untreated adult controls (Fig. 4d, e, black). Previously, studies have shown that interneurons derived from caudal ganglionic eminence (CGE) do not induce cortical plasticity^5,24^. Here, we found that MD-induced reduction in host PV interneuron responsiveness in CGE transplant recipients was equivalent to that in untreated adult control (Fig. 4d, e, magenta). These results suggest that the deprivation-induced reduction in host PV cell responsiveness is specific to MGE transplant recipients.

### Exogenous NRG1 blocks the reduction in binocular PV interneuron responses

The expression of NRG1 in PV interneurons is developmentally regulated^11,15^. Its expression in the visual cortex starts high in early development, remains high during the juvenile critical period but decreases significantly in the adult cortex. The high expression of NRG1 within PV interneurons during the critical period allows these cells to regulate experience-dependent plasticity. We demonstrated previously that exogenous NRG1 prevents the reduction in PV interneuron responses which subsequently blocks cortical plasticity (Fig. 1d, e)^11^. To determine if the reduction in host PV interneuron responses also depended on NRG1/ErbB4 signaling, we treated a second group of MGE transplant recipients with exogenous NRG1 during 1 day of MD. Previous studies have demonstrated that single intraperitoneal injection (I.P.) of this soluble NRG1 (R&D Systems; Human NRG1-beta 1 EGF domain) significantly increases ErbB4 receptor phosphorylation in interneurons^9,11,25^. With repeated peripheral injections (either I.P. or subcutaneous), NRG1 increases the excitability and excitatory inputs to interneurons^9,11^. These results show that exogenous NRG1 crosses the blood-brain barrier when injected peripherally and exerts its effects via ErbB4 receptors on PV interneurons.

Although exogenous NRG1 did not prevent the reduction in the response amplitude of host PV interneurons after MD (Fig. 4e, f, dashed green), we found that it blocked the drop in the percentage of responsive PV interneurons (Fig. 4d, dashed green). Post-hoc immunostaining showed that there was no difference in the overall number of transplanted TdTomato+ cells between the transplant recipients with or without NRG1 treatment (Supplementary Fig. 1f), and >80% of TdTomato+ cells that were positive for PV in both experimental groups (Supplementary Fig. 1g). These results suggest that the differences in the host cell physiology was not due to the differences in the number of transplanted cells between the experimental groups. Furthermore, the overall number of transplanted interneurons was not correlated with the percent change in responsiveness (Supplementary Fig. 1h). Taken together, our results suggest that interneuron transplantation reactivates a developmental program within adult host PV interneurons that re-sensitizes them to visual deprivation.

To further examine how NRG1 affected the host PV interneuron responses, we again performed Ca^2+^ imaging to measure visually evoked responses of PV interneurons after 1 day of MD in transplant recipients treated with NRG1. Time-lapse recording enabled us to track the same host PV interneurons before and after MD and measure deprivation-induced ODP at the single-cell level. Before deprivation, a large fraction of host PV interneurons were visually responsive (80–83%; Fig. 5c), and a little more than 40% of all cells recorded had binocular responses (Fig. 5d). We did not observe any difference in the percentage of responsive cells (Fig. 5c) or binocular responses between all experimental groups (Fig. 5d). Like the excitatory neurons in normal adult mice^26^, there were also PV interneurons that responded only to the contralateral or ipsilateral inputs (Fig. 5b). We determined whether MD differentially affected these groups of host PV interneurons and how exogenous NRG1 counteracted the effect of MD. We found that MD-induced reduction of responsiveness was most pronounced in binocular PV interneurons (Fig. 5e, i), which was prevented in transplant recipients treated with exogenous NRG1 (Fig. 5h, l). With NRG1 treatment, not only did a higher percentage of binocular cells remain binocular, but many previously monocular and unresponsive cells also became binocular. Consequently, the overall percent of responsive binocular PV interneurons remained stable after MD in transplant recipients treated with NRG1. Although many PV interneurons in the untreated adults and CGE transplant recipients also exhibited ODP at the single-cell level (Fig. 5f, g), there was no selective reduction in the responsiveness of binocular cells (Fig. 5j, k).

### Interneuron transplantation accelerates reorganization of binocular excitatory neuron responses

**Fig. 6:**
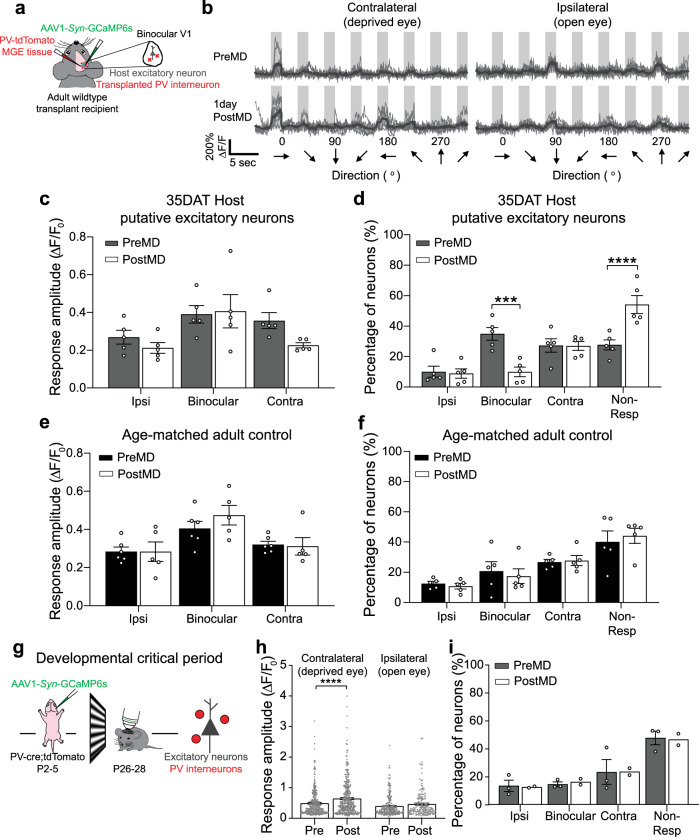
Interneuron transplantation selectively reorganizes binocular responses in host cortex. **a** Schematic of experimental design for transplantation and viral labeling host excitatory neurons. **b** Visually evoked responses before and after MD of example host excitatory neurons at 35DAT. **c** There was no significant change in the response amplitude after MD across all three cell types (*n* = 5 mice; two-way ANOVA with Bonferroni’s multiple comparison test). Ipsi includes neurons that only have significant ipsilateral eye responses, contra includes neurons that only have significant contralateral eye responses. Binocular includes neurons that have both ipsilateral and contralateral responses, but only the dominant eye response amplitude is plotted here. **d** However, percentage of binocular host excitatory neurons was reduced after 1D MD (*n* = 5 mice. ***p = 0.0002; ****p = 0.00009, two-way ANOVA with Bonferroni’s multiple comparison test). **e** No change in the response amplitude of excitatory neurons in adult controls was observed (PreMD: *n* = 6 mice; PostMD: *n* = 5 mice; two-way ANOVA with Bonferroni’s multiple comparison test). **f** The percentage of responsive cells also remained unchanged after 1D MD in age-matched control mice (PreMD: *n* = 6 mice; PostMD: *n* = 5 mice, two-way ANOVA with Bonferroni’s multiple comparison test). **g** Schematic of experimental design for viral labeling excitatory neurons in neonates and Ca^2+^ imaging in critical period animals. **h** During juvenile critical period, the response amplitude of excitatory neurons was significantly increased through the contralateral, deprived eye stimulation (contralateral: PreMD, *n* = 333 cells from 4 mice; PostMD, *n* = 373 cells from 3 mice; Ipsilateral: PreMD, *n* = 247 cells from 3 mice; PostMD, *n* = 173 cells from 2 mice. Contra: ****p = 0.00001; Ipsi: *p* = 0.109, Mann–Whitney *U*-test, two-tailed). **i** However, unlike in the transplant recipients, there was no change in the percentage of responsive binocular neurons after 1D MD in critical period mice. Data in **c**, **d**, **e**, **f**, **h**, and **i** are mean ± SEM. No error bars are shown for PostMD data in **i** since *n* < 3.

During development, MD-induced reduction in the visual responses of PV interneurons results in an enhancement of visual activity in excitatory cells (Fig. 6h)^10,11^. To test whether reduction in host PV inhibition resulted in a similar increase in excitatory neuron response amplitude, we transplanted PV-tdTomato interneuron progenitors from MGE into wild-type C57BL adult mice and then labeled the host cells using non-cre-dependent synapsin-driven AAV1 GCaMP6s (Fig. 6a). In contrast to the developmental critical period, the response amplitude of excitatory neurons remained unchanged after MD (Fig. 6c). When we analyzed all cells and determined whether MD altered the overall responsiveness of excitatory neurons, we were surprised to find that 1 day of MD produced a selective reduction in the percentage of binocular excitatory neurons (Fig. 6d), which was not apparent in the juvenile critical period animals (Fig. 6i) or adult control mice (Fig. 6f). During the critical period, the reorganization of binocular responses usually occurs after a longer period of MD. The fact that the transplant recipients exhibited cortical reorganization after 1 day of MD suggests that interneuron transplantation accelerates cortical plasticity.

### NRG1/ErbB4 signaling within host PV interneurons is necessary for transplant-induced ODP

**Fig. 7:**
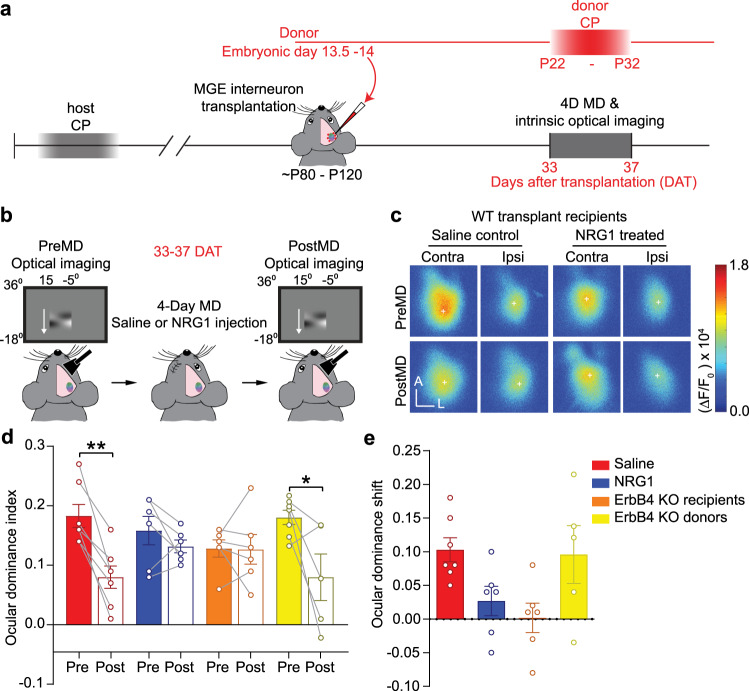
Transplant-induced ODP depends on NRG1/ErbB4 signaling. **a** Experimental timeline. **b** Intrinsic optical imaging was performed to measure ocular dominance plasticity (ODP) in adult transplant recipients around 33–35 days after transplantation (DAT). The wild-type adult recipients received either saline or soluble neuregulin 1 (NRG1) during 4 days of monocular deprivation (MD). **c** Examples of response amplitude before and after 4D MD in WT transplant recipients that received saline injections or were treated with NRG1 during deprivation. Scale bar = 500 µm. **d** Four-day MD resulted in a shift of ocular dominance index (ODI) in transplant recipients that received saline injections. Injections of exogenous NRG1 diminished transplant-induced ODI shift. Interneuron transplantation also failed to reactivate ODP in PV-cre;ErbB4^flx/flx^ adult recipients. However, WT adults that were transplanted with cells from PV-cre;ErbB4^flx/flx^ donor exhibited a significant shift in ODI after 4D MD. ODI is calculated as (Contra response amplitude − Ipsi response amplitude)/(Contra response amplitude + Ipsi response amplitude) (Saline *n* = 7 mice, red; NRG1 *n* = 6 mice, blue; PV-cre;ErbB4^flx/flx^ recipients *n* = 6 mice, orange; PV-cre;ErbB4^flx/flx^ donors PreMD: *n* = 7 mice, PostMD: *n* = 5 mice, yellow. Saline: **p = 0.004; NRG1: *p* = 0.344; PV-cre;ErbB4^flx/flx^ recipients: *p* = 0.844; PV-cre;ErbB4^flx/flx^ donor: *p = 0.03, Mann–Whitney *U*-test, two-tailed). **e** Ocular dominance shift is calculated as (PreMD ODI–PostMD ODI). Ocular dominance shift was reduced in the transplant recipients treated with NRG1 and in PV-cre;ErbB4^flx/flx^ adult recipients (Saline *n* = 7 mice, red; NRG1 *n* = 6 mice, blue; PV-cre;ErbB4^flx/flx^ recipients *n* = 6 mice, orange; PV-cre;ErbB4^flx/flx^ donors *n* = 5, yellow). Data in **d** and **e** are mean ± SEM.

Finally, we asked whether NRG1-mediated reduction in host PV interneuron responsiveness was necessary for transplantation-induced cortical reorganization. Like in the juvenile critical period, we found that transplant-induced OD shift measured using ISOI was prevented in transplant recipients treated with exogenous NRG1 (Fig. 7). In transplant recipients receiving saline injections, 4 days of MD produced a significant reduction in the cortical responses through the deprived eye and thus resulted in a significant shift in OD index (Fig. 7c). In contrast, in transplant recipients treated with exogenous NRG1, the reorganization of cortical responses was blocked (Fig. 7c).

To directly test the possibility that NRG1-dependent plasticity operated through the ErbB4 receptors on the host PV interneurons, we transplanted embryonic MGE tissue into adult recipients in which the ErbB4 receptors were genetically ablated in the host PV interneurons selectively (PV-cre;ErbB4^flx/flx^). We found that interneuron transplantation failed to reactivate cortical plasticity in these mutant mice (Fig. 7d, e). In contrast, ODP appeared normal when we transplanted MGE cells from PV-cre;ErbB4^flx/flx^ mutant embryos into healthy wild-type adults. Altogether, these results further support that the ErbB4 signaling in the host PV interneurons is necessary for transplant-mediated cortical plasticity.

The number of transplanted cells in each group could not account for the differences observed in ODP across different experimental groups (Supplementary Fig. 6). We also did not observe a correlation between the extent of plasticity and the number of transplanted cells (Supplementary Fig. 6e). This was counterintuitive but consistent with Supplementary Fig. 1h, which showed that number of transplanted cells was not correlated with the extent of changes in host interneuron responsiveness. Previous transplantation studies have also shown that the extent of plasticity in the visual cortex^5,7^ and the extent of reactivated fear erasure in the amygdala^27^ are not correlated with the number of transplanted cells. These results could suggest that the pattern of connections formed by the transplanted cells or the changes of the host connectivity induced by transplantation contributes to the reactivation of plasticity more directly than the absolute number of the transplanted cells. However, this merits further investigation.

### Interneuron transplantation increases the level of NRG1 in host PV interneurons

Our in vivo physiology results strongly suggest that NRG1/ErbB4 signaling mediates experience-dependent changes of host PV interneuron activity and transplant-induced host cortical plasticity. We therefore assessed how interneuron transplantation altered the level of NRG1 in the transplant cortex. First, we confirmed that the NRG1 antibody we used could detect the developmental changes of NRG1 in normal PV interneurons (Supplementary Fig. 7a, b). We next immunostained for NRG1 in 35DAT transplant sections (Supplementary Fig. 7c) and found that the expression of NRG1 in the transplant sections was higher than age-matched adult sections. Moreover, we found that the expression of NRG1 in host PV interneurons was also significantly higher in transplanted interneurons (Supplementary Fig. 7e). These results suggest that interneuron transplantation reinstates juvenile NRG1 levels in host PV interneurons.

## Discussion

Using in vivo time-lapse Ca^2+^ imaging, our study reveals the effect of interneuron transplantation on the experience-dependent plasticity of host neurons at single-cell resolution. We demonstrate that MGE interneuron transplantation reinstates a NRG1/ErbB4-dependent reduction of host PV interneurons upon sensory deprivation. First, we show that brief MD results in a rapid reduction in the visual activity of host PV interneurons, to a similar degree as observed in during the developmental critical period (Figs. 1 and 4). Second, the reduction in host PV interneuron responsiveness is blocked by exogenous NRG1 (Figs. 4 and 5). Third, exogenous NRG1 also blocks cortical reorganization that depends on the function of ErbB4 receptors on host PV interneurons (Fig. 7). Taken together, these results suggest that transplanted cells modify host interneurons to create a new critical period for synaptic plasticity.

Our results are surprising because they counter the prevailing idea that transplanted interneurons play a direct role in gating the plasticity of the host circuitry. Even though the transplanted PV interneurons show normal receptive field properties and proper synaptic integration in our study, visual deprivation has little effect on the activity of transplanted PV interneurons. A recent study in which MGE cells from VGAT-mutant embryos fail to induce plasticity in host visual cortex appears to support our initial hypothesis that transplanted PV cell activity regulates the induction of plasticity^6^. As the transplanted VGAT^−/−^ interneurons in that study did not fully mature, reflected in their abnormally developed axonal and dendritic processes, release of the necessary factors for the rejuvenation of NRG1/ErbB4 signaling in the host PV interneurons may have been prevented. In support of our conclusion, another recent transplantation study shows that suppressing the activity of transplanted interneurons using optogenetics after MD does not prevent the expression of transplant-induced ODP^23^. We confirmed this result by selectively suppressing the activity in the transplanted PV cells during MD using DREADD-based chemogenetic approach (Supplementary Fig. 3). Together these results strongly support our idea that host and not transplanted PV cells mediate the reactivated plasticity.

Our study provides a mechanistic insight into transplant-induced cortical reorganization. We highlight the role of NRG1/ErbB4 signaling within a specific cell type—host PV interneurons—in mediating the cortical reorganization.

The mechanisms for NRG1, however, may be different between the normal critical period and transplant-induced critical period. During the developmental critical period, exogenous NRG1 prevents the reduction in both the percentage of responsive PV interneurons and PV cell activity after 1 day of MD and subsequently blocks cortical plasticity^9,11^. In transplant recipients, NRG1 prevents the reduction in the percentage of responsive host PV interneurons but not their response amplitude. The source and downstream signaling of NRG1 remains unclear. Our evidence indicates that NRG1 activates the ErbB4 receptors on the host PV interneurons, but this needs to be further examined since other cell types, including other transplanted non-PV cells, may also express ErbB4 receptors^28^.

Our immunostaining results show that the NRG1 expression within the host PV interneuron is significantly higher than in the age-matched control PV cells and transplanted PV cells at 35DAT. Together with our in vivo physiology results, these results suggest that the interneuron transplantation elevates the level of NRG1 to the host PV interneurons to allow for experience-dependent regulation of cell activity. Future studies can also probe what upstream signals are responsible for reinstating NRG1/ErbB4 signaling in host PV interneurons. High resolution imaging of NRG1 trafficking, e.g., may be informative. It also remains unclear how visual deprivation alters the NRG1 expression. Based on our in vivo physiology results, we predict that MD may selectively decrease the expression of NRG1 within host PV interneurons in transplant recipients.

Our study shows transplanted PV interneurons receive direct long-range inputs, including those from the other hemisphere and lateral geniculate nucleus of the thalamus. The monosynaptic inputs that the transplanted interneurons receive are comparable to the inputs onto endogenous PV interneurons as reported in previous studies^21^. Studies in normal development have demonstrated the importance of thalamic^29–31^ and callosal inputs^32,33^ in ODP, but it is not clear how these long-range inputs to transplanted PV cells contribute to the plasticity of the visual cortex. In future studies, the combination of rabies tracing and in vivo calcium imaging could be used to dissect the role of different long-range inputs in transplant-reactivated cortical plasticity.

In summary, our findings suggest that interneuron transplantation reactivates cortical plasticity by rejuvenating the plasticity program within the host PV interneuron circuit. The mechanism that we describe here resembles parabiosis, in which factors released by young blood cells are thought to restore function to older host blood, muscle, and neural stem cells^34–36^. In our young-to-old neural transplant paradigm, perhaps the transplanted interneurons create a second critical period by introducing rejuvenating factors into the adult host brain. Host PV interneurons may then receive these factors and become re-sensitized to sensory experience. The identification and isolation of these factors may enable development of new therapeutics for treating neurological diseases.

## Methods

### Animals

All protocols and procedures followed the guidelines of the Animal Care and Use Committee at the University of California, Irvine. The following mouse lines were used in this study: B6;129P2-*Pvalb*^*tm1(cre)Arbr/*^J (PV-cre, JAX 017320), B6;129S6-Gt(ROSA)*26Sor*^*tm14(CAG-tdTomato)Hze*/^J (Ai14, JAX 007914), STOCK Slc32a1^*tm2(cre)Lowl*^/J (VGAT-cre, JAX 028862), C57BL/6J (JAX 000664), LSL-R26^TVA-lacZ^ (Rosa-TVA)^37^, and B6N.129-*Gt(ROSA)26Sor*^tm1(CAG-CHRM4*,-mCitrine)Ute^/J (floxed-DREADD, JAX 026219). To visualize either PV interneurons or all GABAergic interneurons, PV-cre or VGAT-cre homozygous mice, respectively, were crossed with cre-dependent red fluorescent Ai14 reporter mice. The embryonic donor tissue used for transplantation was generated either by crossing VGAT-tdTomato or PV-tdTomato homozygous males with C57BL/6J or CD1 wild-type females, or by crossing PV-cre or VGAT-cre homozygous mice with Ai14 homozygous mice. To remove ErbB4 specifically from PV interneurons, the mice homozygous for loxP-flanked (flx) ErbB4 alleles^38^ were first crossed with PV-cre homozygous mice. The resulting PV-Cre^+/−^, ErbB4^flx/+^ mice were crossed with each other to generate PV-Cre^+/+^; ErbB4^flx/flx^ mice, which were used as adult transplant recipients or MGE donors in some of the experiments. To selectively express TVA receptors (avian retroviral receptor, tumor virus A) in PV cells, Rosa-TVA homozygous mice were crossed with PV-cre homozygous animals. For retrograde tracing of transplanted PV cells, we transplanted PV-cre;TVA embryonic tissue into wild-type adult mice. This approach restricts the initial infection of rabies virus to transplanted TVA-expressing PV cells. To selectively express DREADD receptors in the donor PV cells, floxed-DREADD male mice were crossed with PV-cre homozygous female mice to generate PV-cre;DREADD embryos. Refer to Supplementary Data 1 for more details on the experimental groups for each experiment.

### Interneuron transplantation in adult visual cortex

Embryonic days (E) 13.5–14 embryos from timed breeding were used for transplantation. MGE of embryonic forebrain was dissected and suspended in chilled L15 solution (Gibco, 21083027) with 2% (v/v) of DNase I (Roche, 0471672800). All transplantation and subsequent virus injections were performed on the right hemisphere of the animals. For precise targeting, binocular visual cortex (bV1) of adult transplant recipients was mapped using intrinsic optical imaging (described below) at least one week prior to transplantation. The injection sites around bV1 were thinned using a dental drill (Midwest, 78044) and FG1/4 carbide burr to allow the injection micropipette to penetrate. The tissue was loaded into a beveled glass micropipette (75 µm; Wiretrol 5 µl, Drummond Scientific Company) and injected using a custom-made hydraulic manipulator (Narishige, MO-10). The cells were injected at ~650–700 µm below the surface of the brain. Each transplant recipient received 20–30 nl per injection for a total of six to eight injections, all in the same hemisphere. At the end of the procedure, the scalp was sutured using Perma-Hand Silk (Ethicon, J212H) and the animals were returned to the home cage on a heating pad. Refer to Supplementary Data 1 for the genotype and the age of adult transplant recipients, and the type of donor tissue each received.

### Virus injection for calcium imaging

To label neurons in layer 2/3 of binocular visual cortex, adult transplant recipients or age-matched adult controls received either synapsin-driven AAV1-GCaMP6s virus (Penn Vector Core, CS1118; titer: 7 × 10^11^–2 × 10^12^ genome copies (GC)/ml) or cre-dependent synapsin-driven AAV1-GCaMP6s virus (Penn Vector Core, CS1113; titer: 1 × 10^12^ GC/ml). Refer to Supplementary Data 1 for details on the experimental groups. All virus injections were done in only the right hemisphere. Animals were anaesthetized with 2% isoflurane (Patterson Veterinary, 07-893-1389) and received an injection of the analgesic Carprofen (0.8 mg/ml; Rimadyl) before surgery. For adult transplant recipients, virus injection was performed at bV1 between 10–12DAT in the same hemisphere where the transplanted cells were injected. For age-matched adult mice without transplantation, the injection was made at the center of bV1 based on the intrinsic signal map, which was performed at least a week prior. The virus was injected at ~450–600 µm below the surface of the brain (Volume: 2 sites × 200–240 nl/site, rate: 10–12 nl/min) using a beveled glass pipette and a custom-made hydraulic injector.

For labeling cells in critical period mice, the injections were done between postnatal (P) days 2–3. The injections were made at 3–4 mm lateral and 1 mm anterior relative to lambda, roughly where bV1 would be (rate: 30–40 nl/min). The exact location of bV1 in critical period mice was identified using intrinsic optical imaging at P16-18. Mice with off-target virus injection were excluded from the subsequent experiments.

### Headplate and craniotomy

Three to 5 days before in vivo two-photon calcium (Ca^2+^) imaging, a custom-printed headplate was installed and fixed to the right skull using Vetbond (3 M, Vetbond, 1469SB) and dental acrylic (Lang Ortho-Jet Powder and Ortho-Jet Powder Liquid). A cranial window 4–5 mm in diameter was made over primary visual cortex and covered with a glass coverslip of the same size using Vetbond and dental acrylic. Mice received subcutaneous injections of Carprofen to reduce pain and inflammation for up to 3 days after the surgery.

### In vivo Ca^2+^ imaging procedures

A resonant two-photon microscope (Neurolabware, Los Angeles, CA) with a ×16 (Nikon NA = 0.8) objective was used to record Ca^2+^ transients of neurons in layer 2/3 of bV1 (160–350 µm below pia). The laser was set to an excitation wavelength of 910 or 920 nm simultaneously excite GCaMP6s and tdTomato. Emissions were filtered using a 510/84 nm and 607/70 nm BrightLine bandpass filter (Semrock, Rochester, NY). A field of view of ~600 × 250 µm was acquired at 12 Hz (660 lines) using Scanbox acquisition software (Scanbox, Los Angeles, CA). For some recordings, an electrically tunable lens (optotune) was used simultaneously record of multiple depths (at least 25 µm apart for up to 3 depths).

For critical period experiments, some animals were anesthetized during the recording with 0.7% isoflurane and chlorprothixene (Sigma, C1671). The first imaging session started at P26-28. Adult transplant MD experiments were performed in head-fixed, awake mice. The first imaging session started at 33–35DAT, when the transplanted cells reached their normal developmental critical period. Each imaging session consisted of measuring each eye’s responses to drifting gratings in an alternating pattern for a total of two to three sessions for each eye. Because of the sparsity of viral expression, we were able to capture the same PV interneurons in transplant recipients and age-matched adults before and after MD. For excitatory neurons, we located the same fields based on the vasculature.

### One-day monocular deprivation

To examine the effect of 1-day MD, the eye contralateral to the imaging hemisphere was sutured using Perma-Hand Silk (Ethicon, K809H) immediately after the first imaging session. To test the effect of NRG1 on the response properties of PV interneurons, some mice received subcutaneous injections of soluble NRG1 (R&D Systems, 396-HB; 0.5 µg per animal every 8 h) during MD. The suture was carefully removed after 20–30 h. To allow the eye to fully open, animals were returned to their home cage for 30 min after removing the suture. After MD, mice with cataracts, cloudy eyes, and drooping eyelids were excluded from the subsequent experiments and data analysis.

### Visual stimulus conditions for Ca^2+^ imaging

Visual stimuli were generated by custom-written Python code using the PsychoPy 1.8 library^26^. Spherically corrected, full-field drifting sinusoidal gratings were presented in eight orientations and five spatial frequencies (0.03–0.48, logarithmically spaced) at 2 Hz temporal frequency. We also showed a blank condition and a full-field flickering condition. The 42 total stimulus conditions were presented in a different random order for each of the 8 repetitions. Each stimulus was presented for 2 s and followed by 3 s of gray screen for a total of 28 min per imaging session. The stimuli were presented to one eye at a time in an alternating pattern for 2–3 sessions per eye. The stimuli were displayed on an Acer V193 monitor (53 × 33 cm, 60 Hz refresh rate, 20 cd/m^2^ mean luminance), which was positioned 25 cm from the animal.

### Surgical preparation for ISOI

The procedures for ISOI have been previously described^5,11^. ISOI was performed to (a) generate retinotopic maps of bV1, which were then used to guide transplantation and virus injections, and (b) measure OD index in transplant recipients. All imaging was performed through intact skull in animals that were anesthetized with chlorprothixene (0.4 mg/ml) and 0.7% isoflurane. An injection of atropine (Med-Pharmex Inc, 54925-063-10; 1 mg/kg) was also given to dilate the pupil and decrease secretion. To prepare for imaging, the skull was covered with agarose (1.5% w/v in 1× phosphate-buffered saline (PBS)) and a 5 or 10 mm glass coverslip. For hour-long experiments, eye ointment was applied around the coverslip to create a seal and prevent the skull from drying out during imaging. Body temperature was maintained by feedback control heating pad during imaging.

### ISOI in adult transplant recipients

Intrinsic signal images were collected using a custom-designed macroscope (Nikon 135 × 50 mm lenses) equipped with a Dalsa 1M30 CCD camera. First, a green (530 nm) light-emitting diode (LED) was used to visualize and capture an image of the surface vasculature. Then the camera was focused ~600 µm beneath the pia surface, and a red (617 nm) LED light was used to acquire the intrinsic signal. For adult MD experiments, all mice underwent both PreMD and PostMD imaging sessions, three to four times each. Each imaging session consisted of measuring the responses through each eye in an alternating pattern for a total imaging duration of 30–40 min.

### Four-day monocular deprivation

For ODP experiments, the eye contralateral to the imaging hemisphere was sutured immediately after the first day of imaging and stayed closed for 4 days, which has been previously shown to effectively induce cortical plasticity^5,11^. To examine the effect of exogenous NRG1 on ODP in transplant recipients, some recipients were injected with either saline or NRG1 (0.5 µg per animal every 8 h) during MD. For the WT adult mice that received donor cells from PV-cre;DREADD embryos, 2 injections of CNO (Enzo Life Sciences, BML-NS105-0005; 5 mg/kg dissolved in 0.9% sterile saline and 1% dimethyl sulfoxide) were given at 12 h interval during the 4 days of MD.

### Visual stimulus conditions for ISOI

The MATLAB Psychophysics Toolbox extensions was used to generate visual stimuli^39,40^. Visual stimuli consisted of contrast modulating sweeping noise. The display of the stimuli was restricted to −5° to +15° visual field azimuth and −18° to +36° visual field elevation. Each recording trial consisted of five-minute presentations of stimuli at 0° and 180°. The stimuli were displayed on an Acer V193 monitor (53 × 33 cm, 60 Hz refresh rate, 20 cd/m^2^ mean luminance), which was positioned 25 cm from the animal.

### Retrograde viral tracing using rabies virus

Retrograde viral tracing using glycoprotein-deleted rabies virus was previously described^18,19^. Three weeks after the transplantation of PV-cre;TVA tissue, transplant recipients were anesthetized under 1.5–2% isoflurane and injected with Carprofen before surgery. For labeling the inputs onto normal PV interneurons, we performed retrograde tracing in PV-cre;TVA animals a few days during the juvenile critical period (P29–P31). A pulled glass pipette (tip diameter, ~30 μm) was loaded with virus and then lowered into the brain (400 and 600 μm below the pial surface). A Picospritzer (General Valve, Hollis, NH) was used to deliver the virus. The helper virus (pAAV-Ef1a-DIO-H2B-GFP-2A-oG-WPRE-hGH; Addgene, Plasmid 74289; 1.5 × 10^13^ GC/ml; 2 sites × 200 nl/site) was injected at bV1 at a rate of 8–10 nl/min. To prevent backflow of virus, the pipette remained in the brain for 5 min after completion of the injection. After the procedure, mice were allowed to recover in their home cages on a heating pad. Three weeks after the AAV injection, the RG-deleted rabies virus (EnvA-SADΔG-DsRed rabies, 200 nl, ∼2 × 10^9^ GC/ml) was injected into the same location of the previous helper virus injection in bV1. The rabies virus was allowed to incubate for 9–10 days before the animals were perfused for tissue processing.

### Immunohistochemistry

The mice were perfused with chilled 1× PBS and 4% paraformaldehyde (PFA). The brains were post-fixed overnight and cryoprotected with 30% sucrose. The 50 µm sections containing primary visual cortex were prepared using a microtome (Microm, HM450). For NRG1 immunostaining experiments, the sections were cut at 25 µm.

Immunohistolochemistry of free-floating sections was previously described^11^. Sections were first permeabilized with 0.3% Triton-X in PBS and then blocked with 0.3% Triton-X in PBS containing 5% normal goat serum (Vector Laboratories, S-1000) and 10% bovine serum albumin (Sigma, A7906). After 48–72 h of incubation in primary antibodies for 48–72 h at 4 °C, sections were rinsed with 1× PBS, and incubated overnight with secondary antibodies.Sections were then mounted with Fluoroshield with DAPI (Millipore-Sigma, F6057), and cover-slipped. To quantify PV + cells in the transplant brains, selected sections were stained with PV antibody (Sigma, P3088, Mouse, 1 : 500), followed with Alexa Fluor-conjugated secondary (Invitrogen, A21240, Alexa 647-conjugated goat anti-mouse, 1 : 1000). To identify the starter cells and determine the cell identities of rabies-infected DsRed-positive cells in rabies-infected brains, sections containing V1 were co-stained with GFP antibody (Aves, GFP-1010, Chicken, 1 : 500) and GABA (Sigma, 2052, rabbit, 1 : 1000) antibodies. Alexa 647-conjugated goat anti-rabbit (Invitrogen, A21245, or Jackson ImmunoResearch, 111-605-003, 1 : 1000), and Alexa 488-conjugated goat anti-chicken (Invitrogen, A11039, 1 : 1000) secondary antibodies were used to visualize the staining. For NRG1 immunostatining, the sections were incubated in primary antibody for up to 6 days. Negative control sections without primary antibodies for each age group were included for every batch of experiments. To stain NRG1 in the normal PV-tdTomato sections, only NRG1 primary antibody (ThermoFisher, PA5-78627, rabbit, 1 : 250) was used. To stain for NRG1 in both the host and transplanted PV cells, the transplant sections were co-stained with PV and NRG1 antibodies. Alexa 488-conjugated goat anti-mouse (Invitrogen, A11001, 1 : 1000) and Alexa 647-conjugated goat anti-rabbit (Jackson ImmunoResearch, 1 : 500).

### iDISCO+ whole brain clearing

All samples were cleared as previously described^41^. For full protocol details and solution reagents, refer to idisco.info.

#### Pretreatment

Briefly, fixed transplant brains were washed three times in PBS for 1 h to remove PFA. Samples were then subjected to a series of 1 hr MeOH dehydration steps of increasing concentration from 20% to 100% at room temperature (RT). After overnight fixation in 2 : 1 dicholoromethane (DCM, Sigma, 270997) : MeOH, samples were washed twice with 100% MeOH. Samples were briefly incubated at 4 °C, then rocked overnight in freshly made, pre-chilled 1 : 5 30% H_2_O_2_ : 100% MeOH bleaching solution at 4 °C. Upon removal from 4 °C, samples were subjected to a series of 1 h MeOH rehydration steps of decreasing concentration from 80% to 0% at RT.

#### Immunolabeling

Samples were washed twice in PTx.2 at RT, followed by permeabilization for 2 days at 37 °C. Samples were again washed twice in PTx.2 at RT, then blocked for 3 days at 37 °C. To amplify the host AAV1-GCaMP6s and transplanted PV-tdTomato signals, samples were washed twice in PTwH at RT and incubated for 6 days at 37 °C with 1 : 500 chicken anti-GFP (Aves Labs, GFP-1020) and 1 : 400 rabbit anti-RFP (Rockland, 600-401-379), respectively. Samples were then washed in PTwH for increasing durations of 0 min to 2 h for the first day, 2 h each for the second. Following a secondary incubation for 6 days at 37 °C with 1 : 500 donkey anti-chicken Alexa 647 (Jackson ImmunoResearch, 703-605-155) and 1 : 400 donkey anti-rabbit Alexa 568 (Abcam, ab175692), samples were washed using the same post-primary protocol.

#### Clearing

Samples were subjected to a final series of 1 h MeOH dehydration steps of increasing concentration from 20% to 100% at RT. After another overnight fixation in 2 : 1 DCM : MeOH, samples were incubated twice for 15 min with 100% DCM. To render the tissue optically clear, samples were lastly incubated with dibenzyl ether (DBE, Sigma, 108014) at RT.

### Light-sheet microscopy

Equipped with an s-CMOS camera and multi-view imaging, a light-sheet fluorescence microscope (Zeiss Z.1) was used to image cleared transplant brains as whole brains. Cleared brains were first mounted on custom three-dimensional (3D) printed suspension clamps and submerged in a large custom chamber (Translucence) filled with DBE. Using two illumination objectives (EC Plan-Neofluor ×5/0.16), cleared brains were excited by LED lasers (561-50 and 638-75, SBS LP 640) and emissions collected by a collared (*n* = 1.45) detection objective (LSFM ×5/0.1). Tile scan configurations were calculated using an external multi-view-setup program (Zeiss). Sixteen-bit resolution scans were captured at ×1 zoom magnification, with laser power (561: 15%, 638: 20%) and exposure time (70 ms) optimized for clarity and high signal-to-noise ratio. Scans retained a constant interval (5.324 μm), overlap (20%), and delay (4 sec) between *z*-stack tiles.

### 3D image reconstruction

Overlapping regions between tiles were used for manual realignment and creation of stitched, multi-dimensional image stacks via Arivis. Image stacks were post-processed in Imaris, applying a gamma correction (0.8) to reduce auto-fluorescence intensity. Orthogonal slice animations were also generated using Imaris (Supplementary Movie 1).

### Calcium imaging data analysis

Custom-written Python scripts were used to compute the fluorescence signal of each cell body by subtracting the average neuropil signal from the signal of the soma. Each cell’s response to each stimulation period was normalized to the baseline value preceding stimulus presentation (Δ*F*/*F*_0_). A neuron was included in the following analysis when its responses to each stimulation across all spatial frequencies reached significance when compared against the blank condition (one-way analysis of variance (ANOVA), *p* < 0.01). At each spatial frequency, the cell’s response to a given orientation *θ* was defined as the average response across the eight repeats of that orientation. The peak spatial frequency was defined as the frequency that produces the maximum average response, and this response was chosen as *F*(*θ*).

The cell’s preferred orientation at its peak spatial frequency was then determined as follows:

1 $$\theta _{\mathrm{pref}} = \frac{{{\sum} {F(\theta )e^{2i\theta }} }}{{2{\sum} {F(\theta )} }}$$

The fitted response *R(θ)* was then determined by fitting *F(θ)* to a sum of two Gaussians centered on *θ*_pref_ and *θ*_pref_ + *π*, with different amplitudes and equal width, and a constant baseline. The amplitude of the fitted response at the preferred orientation was *R(θ*_pref_*)*, and this value is reported as a cell’s response amplitude in the figures and text. Any cells with a response amplitude below 5% were considered not responsive.

The cell orientation selectivity index was determined using the circular variance method based on the following equation^26,42^:

2 $${\mathrm{ISO}} = \sqrt {\left( {\mathop {\sum}\nolimits_i {\left( {F\left( {\theta _i} \right)} \right) \ast \sin \left( {2\theta _i} \right)} } \right)^2 + \left( {\mathop {\sum}\nolimits_i {\left( {F\left( {\theta _i} \right)} \right) \ast \cos \left( {2\theta _i} \right)} } \right)^2/\mathop {\sum}\nolimits_i {F\left( {\theta _i} \right)} }$$

Time-lapse imaging of PV interneurons in transplant recipients and untreated adults allowed us to track the same cells before and after MD. For calculating the percent change in response amplitude (Fig. 3e, f), only cells that were responsive during both Pre- and PostMD imaging sessions were included. We first calculated percent change for individual cells, and then calculated the average for each field. The actual data points plotted are therefore the average response amplitude per field (Fig. 3e, f). For calculating the percent difference in responsiveness for each field, we included the same cells before and after MD (Fig. 3d). Each animal contributed two to five fields.

The ODI for each cell was calculated as (*C* − *I*)/(*C* *+* *I*), where *C* is *R*(*θ*_pref_) for the contralateral eye and I is *R*(*θ*_pref_) for the ipsilateral eye. Contralaterally dominated neurons have an ODI value of 1, and these neurons are labeled as Contra throughout the paper. Ipsilaterally dominated neurons have an ODI value of −1, and they are labeled as Ipsi. Binocular neurons have ODI between −1 and 1. In cases where no significant response was detected for one eye, response amplitude for that eye was set to 0. Non-responsive cells are those neurons that showed no significant response to either eye. In Fig. 6c, e, the response amplitude for the dominant eye responses was plotted as Binocular. The percent responsiveness of each group is calculated as the number of responsive cells in that group divided by the total number of cells including both responsive and non-responsive cells.

### ISOI data analysis

Custom-written Matlab scripts were used to generate amplitude and phase maps of cortical responses using Fourier analysis as described previously^5,7,11,43,44^. The Fourier map was then smoothed with a 5 × 5 Gaussian kernel to compute the final amplitude map. The phase map was normalized by subtracting the phase of the cortical responses at 180° from the phase at 0°, to remove hemodynamic delay. Maps of absolute retinotopic phase are shown in terms of visual angle relative to the center of the monitor. Phase maps are then overlaid on top of vasculature image to facilitate the targeting of bV1 in other surgical and imaging procedures.

The ODI for each animal was calculated as (*C* − *I*)/(*C* *+* *I*), where *C* is the average response amplitude for the contralateral eye and *I* is the average response amplitude for the ipsilateral eye across three or four repetitions. OD shift is used as a measure for cortical plasticity and is calculated by subtracting PreMD ODI from PostMD ODI.

### Immunohistochemistry image analysis

Immunostained sections were imaged using a confocal microscope (Leica SP8, ×20 Objective, Leica Microsystem, Germany). LAS X image processing software (Leica Microsystem) was used to perform image tiling and acquire z-stack. To count the transplanted cells in bV1, sections were sampled at 300 µm intervals. The tdTomato red fluorescence was bright enough to count the transplanted cells without amplification. The percentage of transplanted tdTomato cells that co-expressed PV was calculated by dividing the number of PV+/tdTomato+ neurons by the total number of tdTomato+ cells. The percentage of host PV interneurons was calculated by dividing the number of PV+/GCaMP6+ co-labeled cells by the total number of GCaMP6+ cells. The GCaMP6+ transplanted cells were excluded. In rabies-infected V1, the starter cells expressed DsRed (rabies), nuclear GFP, and GABA. No amplification was needed to identify DsRed+ cells. The cells that only expressed DsRed and GABA were categorized as presynaptic GABAergic neurons. We counted GABA-negative DsRed-positive cells as putative presynaptic excitatory neurons.

For quantifying the NRG1 expression intensity in PV interneurons, cell bodies were selected based on tdTomato or PV expression in the binocular V1. The background fluorescence was determined for each stained section. Corrected total fluorescence per cell^11^ in arbitrary units was calculated as Corrected Total Cell Fluorescence (CTCF) = Integrated Density − (Area of selected cell × Mean fluorescence of background fluorescence). The mean CTCF value was then calculated for each section and normalized to the mean values calculated for the normal adult sections (Supplementary Fig. 7).

### Statistics and reproducibility

Experiments were replicated successfully with multiple animals. Number of replications and *N*’s (number of animals, cells or imaging fields) are indicated in the figure legends. Either parametric (*t*-test with Welch’s correction, Brown–Forsythe one-way ANOVA test followed by Dunnett’s T3 multiple comparison test, ordinary one-way ANOVA) or non-parametric (two-way ANOVA followed by Bonferroni multiple comparison test, Mann–Whitney *U*-test, Wilcoxon signed-rank test, Kruskal–Wallis one-way ANOVA followed by Dunn’s multiple comparison test) statistics was performed. Normality of the data was tested using D’Agostino–Pearson normality test and non-parametric statistics was performed when the data was not normally distributed. In addition, linear regression and correlation analysis and Fisher’s exact test were also performed where appropriate. Refer to Supplementary Data 2 for detail.

### Reporting summary

Further information on research design is available in the Nature Research Reporting Summary linked to this article.

## Supplementary information

Supplementary Information

Description of Additional Supplementary Files

Supplementary Data 1

Supplementary Data 2

Supplementary Movie 1

Reporting Summary

## Data Availability

Source data are provided with this paper and its Supplementary Information files. Additional information is available from the corresponding author upon reasonable request. Source data are provided with this paper.

## Source data

Source Data

## Code availability

The custom-written MATLAB and python scripts used for data collection and processing have been described in previously published manuscripts from the lab^26,29^. The code used for generating visual stimulus and for data analysis is available from the corresponding author upon reasonable request.
