# Supplementary material for: Host interneurons mediate plasticity reactivated by embryonic inhibitory cell transplantation in mouse visual cortex
Source: Nat Commun. 2021 Feb 8;12:862. doi: 10.1038/s41467-021-21097-4 (PMC7870960; doi:10.1038/s41467-021-21097-4)
Supplement: Supplementary file 2 — Description of Additional Supplementary Files [file 41467_2021_21097_MOESM2_ESM.docx]

**Description of Additional Supplementary Files**

File Name: Supplementary Movie

Description: An example of a cleared transplant brain. The animation displays 3D reconstructed horizontal slices, moving from ventral to dorsal surface of the cortex. Green is GCaMP6 labelled V1 host PV interneurons, and red is transplanted PV interneurons.

File Name: Supplementary Data 1

Description: Experimental groups

File Name: Supplementary Data 2

Description: Summary statistics
