# Supplementary material for: Host interneurons mediate plasticity reactivated by embryonic inhibitory cell transplantation in mouse visual cortex
Source: Nat Commun. 2021 Feb 8;12:862. doi: 10.1038/s41467-021-21097-4 (PMC7870960; doi:10.1038/s41467-021-21097-4)
Supplement: Supplementary file 6 — Reporting Summary [file 41467_2021_21097_MOESM6_ESM.pdf]

nature research

Corresponding author(s): Sunil P. Gandhi

Last updated by author(s): Dec 19, 2020

Reporting Summary

Nature Research wishes to improve the reproducibility of the work that we publish. This form provides structure for consistency and transparency in reporting. For further information on Nature Research policies, see Authors & Referees and the Editorial Policy Checklist.

Statistics

For all statistical analyses, confirm that the following items are present in the figure legend, table legend, main text, or Methods section.

n/a	Confirmed	
☐	☒	The exact sample size (*n*) for each experimental group/condition, given as a discrete number and unit of measurement
☐	☒	A statement on whether measurements were taken from distinct samples or whether the same sample was measured repeatedly
☐	☒	The statistical test(s) used AND whether they are one- or two-sided<br>*Only common tests should be described solely by name; describe more complex techniques in the Methods section.*
☒	☐	A description of all covariates tested
☐	☒	A description of any assumptions or corrections, such as tests of normality and adjustment for multiple comparisons
☐	☒	A full description of the statistical parameters including central tendency (e.g. means) or other basic estimates (e.g. regression coefficient) AND variation (e.g. standard deviation) or associated estimates of uncertainty (e.g. confidence intervals)
☐	☒	For null hypothesis testing, the test statistic (e.g. *F*, *t*, *r*) with confidence intervals, effect sizes, degrees of freedom and *P* value noted<br>*Give P values as exact values whenever suitable.*
☒	☐	For Bayesian analysis, information on the choice of priors and Markov chain Monte Carlo settings
☒	☐	For hierarchical and complex designs, identification of the appropriate level for tests and full reporting of outcomes
☐	☒	Estimates of effect sizes (e.g. Cohen's *d*, Pearson's *r*), indicating how they were calculated

Our web collection on statistics for biologists contains articles on many of the points above.

Software and code

Policy information about availability of computer code

Data collection	Brain section images were acquired using imaging acquisition software Leica Application Suite X (LAS X, https://www.leica-microsystems.com). Cell counting and immunohistochemistry quantification were performed offline using LAS X offline software (Leica, 2018).<br>Whole brain 3D image reconstruction was performed using Arivis (https://www.arivis.com/; Vision 4D x64, version 3.1) and Imaris x64 (https://imaris.oxinst.com/; version 9.5.0)<br>Visual stimuli for calcium imaging imaging were generated by custom-written Python code using the PsychoPy 1.8 library.<br>Two-photon calcium imaging data was collected using a commercial software Scanbox (https://scanbox.org/2014/03/13/welcome-to-scanbox/; version 2 and 3)<br>For intrinsic optical signal imaging, an image acquisition package gVision in MATLAB (MathWorks; version 2016a) was used to acquire images and stream to disk (https://github.com/guslott/gvision).
Data analysis	Custom-built analysis software was used to draw cell ROIs, extract calcium fluorescence traces, and determine peak response amplitude and tuning properties (Huh et al., 2020; Salinas et al., 2017).<br>For intrinsic signal optical imaging, custom-written MATLAB code was used to generate phase maps and determine the visual cortical responses (Davis et al., 2015; Sun et al., 2016).<br>All statistical tests were performed in GraphPad Prism 7 or 8 (https://www.graphpad.com/scientific-software/prism/)<br>All custom-written code is available from the corresponding author upon reasonable request.

For manuscripts utilizing custom algorithms or software that are central to the research but not yet described in published literature, software must be made available to editors/reviewers. We strongly encourage code deposition in a community repository (e.g. GitHub). See the Nature Research guidelines for submitting code & software for further information.

Data

Policy information about availability of data

All manuscripts must include a data availability statement. This statement should provide the following information, where applicable:

- Accession codes, unique identifiers, or web links for publicly available datasets
- A list of figures that have associated raw data
- A description of any restrictions on data availability

The data supporting the findings of this study are available within the paper and reported in Source Data file. Additional information is available from the corresponding author upon reasonable request.

Field-specific reporting

Please select the one below that is the best fit for your research. If you are not sure, read the appropriate sections before making your selection.

☒ Life sciences ☐ Behavioural & social sciences ☐ Ecological, evolutionary & environmental sciences

For a reference copy of the document with all sections, see nature.com/documents/nr-reporting-summary-flat.pdf

Life sciences study design

All studies must disclose on these points even when the disclosure is negative.

Sample size	Sample sizes (i.e. number of cells, number of imaging fields, number of sections, and number of animals) were approximated based on previous transplantation studies (Davis et al., 2015; Figueroa-Velez et al., 2017) and other works from our and collaborator's labs (Huh et al., 2020; Salinas et al., 2017; Sun et al., 2016).
Data exclusions	For both intrinsic and calcium imaging experiments, animals with droopy or swollen eyelids, cataract, or sutures that did not stay intact after monocular deprivation were excluded from further experiments. In some cases, data gathered before monocular deprivation were still included in the final data analysis. For comparison of the same cells, only animals that had both before and after deprivation recordings were included.<br>Post-hoc histological analysis was performed to confirm the presence of transplanted cells in transplant recipients. Animals with no visible cells as indicated by TdTomato fluorescence were excluded in the data analysis.<br>For Ca2+ imaging, animals were excluded if the virus injection was off-target, or had damage to the brain due to surgeries or bone growth over the cranial window.<br>For rabies viral tracing experiments, only animals that expressed starter cells in the primary visual cortex were included.
Replication	All experiments were replicated successfully with multiple mice. The number of mice used for each experiment is stated in the manuscript.
Randomization	Mice were assigned to either saline or neuregulin-1 treatment groups randomly after Pre-dperivation imaging session.
Blinding	For intrinsic signal imaging of transplant recipients, the experimenter was blind to the saline vs. NRG1 treatment, and blind during the data analysis.<br><br>For calcium imaging of transplant recipients, the experimenter was not blind to the experiment condition during imaging since the fluorescence and appearance of the transplanted cells would reveal the experimental condition of the animal. To mitigate the bias, for some of the imaging experiments, imaging and data analysis were performed by different experimenters.

Reporting for specific materials, systems and methods

We require information from authors about some types of materials, experimental systems and methods used in many studies. Here, indicate whether each material, system or method listed is relevant to your study. If you are not sure if a list item applies to your research, read the appropriate section before selecting a response.

Materials & experimental systems

n/a	Involved in the study	
☐	☒	Antibodies
☒	☐	Eukaryotic cell lines
☒	☐	Palaeontology
☐	☒	Animals and other organisms
☒	☐	Human research participants
☒	☐	Clinical data

Methods

n/a	Involved in the study	
☒	☐	ChIP-seq
☒	☐	Flow cytometry
☒	☐	MRI-based neuroimaging

Antibodies

Antibodies used	All antibodies used in the current study have been described in detail in the Methods. The primary antibodies include:<br>Mouse anti-Parvalbumin, (Sigma, Cat.# P3088, 1:500 dilution)<br>Chicken anti-GFP (Aves, Cat.# GFP-1010, 1:500, dilution)<br>Rabbit anti-GABA (Sigma, Cat.# 2052, 1:1000 dilution)<br>Rabbit anti-RFP (Rockland, Cat.# 600-401-379, 1:400 or 1:500 dilution)<br>Rabbit anti-NRG1 (ThermoFisher, Cat. # PA5-78627, 1:250 dilution)<br><br>The secondary antibodies include:<br>Goat anti-Mouse Alexa 488 (Invitrogen, Cat. # A11001, 1:1000)<br>Goat anti-Mouse Alexa 647 (Invitrogen, Cat.# A21240, 1:1000)<br>Goat anti-Chicken Alexa 488 (Invitrogen, Cat.# A11039, 1:1000)<br>Goat anti-Rabbit Alexa 647 (Invitrogen, Cat.# A21245, 1:1000)<br>Goat anti-Rabbit Alex 647 (Jackson ImmunoResearch, Cat.# 111-605-003, 1:500 or 1:1000)<br>Donkey anti-Chicken Alexa 647 (Jackson ImmunoResearch Laboratories, Cat.# 703-605-155, 1:500)<br>Donkey anti-Rabbit Alexa 568 (Abcam, Cat.# ab175692, 1:400)
Validation	All of the antibodies used in our study are commercially available.<br><br>For mouse anti-PV monoclonal antibody, Sigma Aldrich provides validation information as follows:<br>"Monoclonal Anti-Parvalbumin (mouse IgG1 isotype) is derived from the PARV-19 hybridoma produced by the fusion of mouse myeloma cells and splenocytes from an immunized mouse. Purified frog muscle parvalbumin was used as the immunogen. The isotype is determined using Sigma ImmunoTypeTM Kit (Product Code ISO-1) and by a double diffusion immunoassay using Mouse Monoclonal Antibody Isotyping Reagents (Product Code ISO-2).<br><br>For chicken anti-GFP polyclonal antibody, Aves Labs provides validation information as follows<br>"Antibodies were analyzed by western blot analysis (1:5000 dilution) and immunohistochemistry (1:500 dilution) using transgenic mice expressing the GFP gene product. Western blots were performed using BlokHen® (Aves Labs) as the blocking reagent, and HRP-labeled goat anti-chicken antibodies (Aves Labs, Cat. #H-1004) as the detection reagent. Immunohistochemistry used tetramethyl rhodamine-labeled anti-chicken IgY"<br><br>For rabbit anti-GABA antibody, Sigma Aldrich provides validation information as follows:<br>Anti-GABA is produced in rabbit using "GABA-BSA as the immunogen. The antibody is isolated from antiserum by immunospecific methods of purification. Antigen specific affinity isolation removes essentially all rabbit serum proteins, including immunoglobulins which do not specifically bind to GABA.<br>Anti-GABA shows positive binding with GABA, and GABA-KLH in a dot blot assay, and negative binding with BSA."<br><br>For rabbit anti-RFP antibody, Rockland provides validation information as follows:<br>"This product was prepared from monospecific antiserum by immunoaffinity chromatography using Red Fluorescent Protein (Discosoma) coupled to agarose beads followed by solid phase adsorption(s) to remove any unwanted reactivities. Expect reactivity against RFP and its variants: mCherry, tdTomato, mBanana, mOrange, mPlum, mOrange and mStrawberry. Assay by immunoelectrophoresis resulted in a single precipitin arc against anti-Rabbit Serum and purified and partially purified Red Fluorescent Protein (Discosoma). No reaction was observed against Human, Mouse or Rat serum proteins. ."<br><br>For rabbit anti-NRG1 antibody, ThermoFisher provides validation information as follows:<br>"Western blot analysis of NRG1 in various tissue extracts using 50 µg of protein. Samples were separated with 7.5% SDS-PAGE and incubated with NRG1 polyclonal antibody (Product # PA5-78627) using a dilution of 1:1000."<br>"Immunohistochemistry analysis of NRG1 in paraffin-embedded adult mouse hippocampus. Sample was incubated with NRG1 polyclonal antibody (Product # PA5-78627) using a dilution of 1:250 followed by DAPI (blue)."

Animals and other organisms

Policy information about studies involving animals; ARRIVE guidelines recommended for reporting animal research

Laboratory animals	The following mouse lines were used in this study: B6;129P2-Pvalbtm1(cre)Arbr/J (PV-cre, JAX 017320), B6;129S6-Gt(ROSA)26Sortm14(CAG-tdTomato)Hze/J (Ai14, JAX 007914), STOCK Slc32a1tm2(cre)Lowl/J (Vgat-ires-cre, JAX 028862), C57BL/6J (JAX 000664), LSL-R26TVA-lacZ (Rosa-TVA) (26), B6N.129-Gt(ROSA)26Sortm1(CAG-CHRM4*,-mCitrine)Ute/J (floxed-DREADD, JAX 026219). To visualize either parvalbumin (PV) interneurons or all GABAergic interneurons, PV-cre or VGAT-cre homozygous mice, respectively, were crossed with cre-dependent red fluorescent Ai14 reporter mice. Embryonic tissues used for transplantation were either PV-tdTomato or VGAT-tdTomato-positive. PV-Cre+/+; ErbB4flx/flx mice were used as adult transplant recipients in some of the intrinsic optical imaging experiments. To selectively express TVA receptors (avian retroviral receptor, tumor virus A) in PV cells, Rosa-TVA homozygous mice were crossed with PV-cre homozygous animals. For retrograde tracing of transplanted PV cells, we transplanted PV-cre;TVA embryonic tissue into wildtype adult mice. To selectively express DREADD receptors in the donor PV cells, floxed-DREADD male mice were crossed with PV-cre homozygous female mice to generate PV-cre;DREADD embryos. Experimental subjects included both males and females. Refer to Table S1 for more details on the experimental groups for each experiment.
Wild animals	No wild animals were used in this study.
Field-collected samples	No field-collected samples were in this study.

Ethics oversight	All protocols and procedures followed the guidelines of the Animal Care and Use Committee at the University of California, Irvine.

Note that full information on the approval of the study protocol must also be provided in the manuscript.
