# Supplementary material for: Host interneurons mediate plasticity reactivated by embryonic inhibitory cell transplantation in mouse visual cortex
Source: Nat Commun. 2021 Feb 8;12:862. doi: 10.1038/s41467-021-21097-4 (PMC7870960; doi:10.1038/s41467-021-21097-4)
Supplement: Supplementary file 1 — Supplementary Information [file 41467_2021_21097_MOESM1_ESM.pdf]

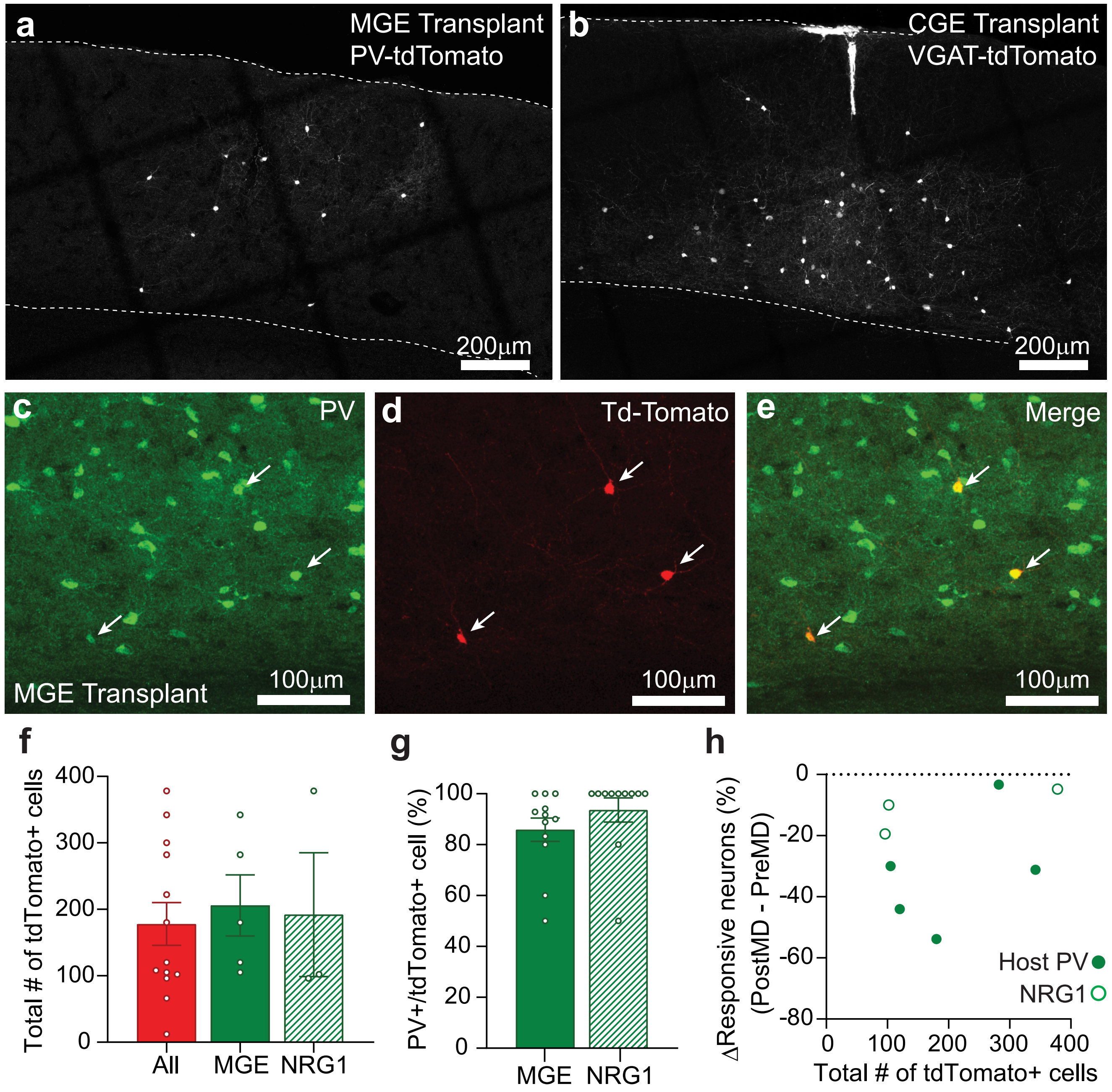
a
MGE Transplant
PV-tdTomato
200µm
b
CGE Transplant
VGAT-tdTomato
200µm
c
PV
MGE Transplant
100µm
d
Td-Tomato
100µm
e
Merge
100µm
f
Total # of tdTomato+ cells
400
300
200
100
0
All
MGE
NRG1
g
PV+/tdTomato+ cell (%)
100
80
60
40
20
0
MGE
NRG1
h
ΔResponsive neurons (%)
(PostMD - PreMD)
0
-20
-40
-60
-80
0
100
200
300
400
Total # of tdTomato+ cells
Host PV
NRG1

Supplementary Figure 1. Transplanted cells express marker of mature PV interneurons. a, Example coronal section from a PV-tdTomato MGE recipient 35 days after transplantation (DAT). Transplanted cells dispersed across cortical layers. Post-hoc confirmation of transplanted PV interneurons was performed in 13 transplant recipients after the completion of *in vivo* imaging experiments. **b,** Example coronal section from a VGAT-tdTomato CGE recipient 35 DAT. Transplanted cells dispersed but appeared biased to the deeper layers. Scale bar = 200 µm. Presence of transplanted CGE cells was confirmed in 2 transplant recipients *post-hoc*. **c-e,** Immunostaining of parvalbumin (PV) was performed in 5 different MGE transplant recipients after the completion of *in vivo* imaging experiments. **c,** Example coronal section from a PV-tdTomato MGE recipient at 35DAT stained for parvalbumin (green). Scale bar = 100 µm. **d,** In the same section as **c**, transplanted PV cells expressed red fluorescent protein tdTomato (red). **e,** Transplanted PV-tdTomato cells (red) stained for parvalbumin (yellow). **f,** Estimated number of transplanted PV-tdTomato cells in all MGE transplant recipients (n = 13 mice, red, including "MGE" and "NRG1" groups). MGE includes transplant recipients for which host PV cells were imaged in Ca^{2+} imaging experiments at 35DAT (n = 5 mice). NRG1 includes transplant recipients for which host PV cells were imaged but also treated with excess soluble NRG1 (n = 3 mice; see also Figures 4 and 5). There was no difference between the estimated total number of transplanted PV-tdTomato cells between MGE and NRG1 treated groups ($p = 0.571$, two-tailed, Mann-Whitney U test). Data are mean ± SEM. **g,** Quantification of transplanted PV-tdTomato cells coexpress PV. More than 80% of transplanted cells co-expressed PV in both MGE (n = 12 sections from 3 mice) and NRG1 groups (n = 11 sections from 2 mice). Data are mean ± SEM. **h,** There is no correlation between the total number of transplanted PV-tdTomato cells and the change in responsiveness observed in MGE or NRG1 treated recipients ($R^2 = 0.112$).

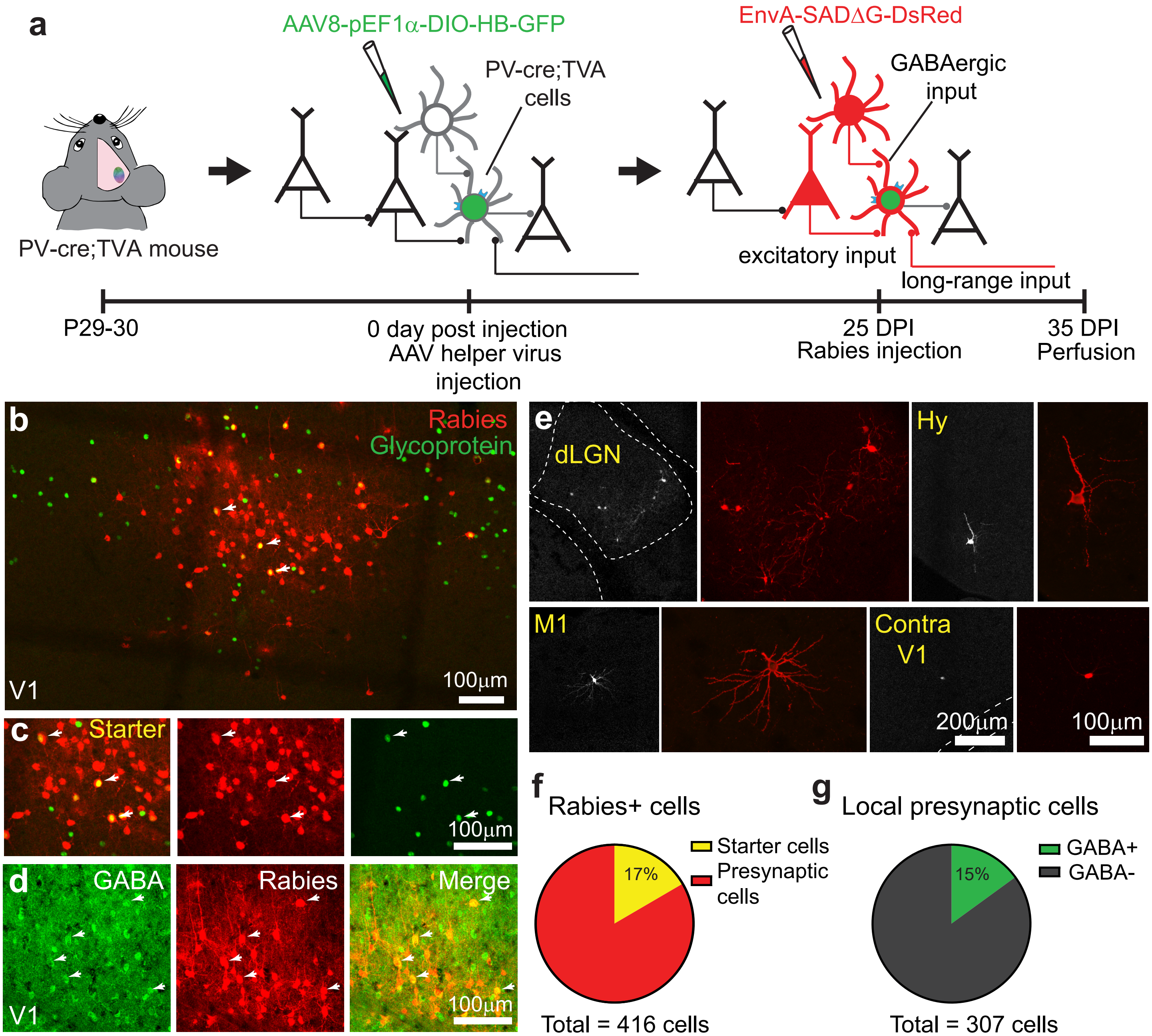

Supplementary Figure 2. Local and long-range inputs to PV interneurons in adult PV-TVA mice. a, Experimental design. **b,** Identification of starter PV cells by co-expression of rabies glycoprotein (green) and rabies virus (red). scale bar = 100 μm. 50-μm max projection. **c,** Magnified images of the starter cell in b, Scale bar = 100 μm. **d,** PV cells received both GABAergic (yellow) and putative excitatory inputs (red) from the host cortex. Scale bar = 100 μm. **e,** PV cells received long-range inputs from various brain regions. Black and white images, scale bar = 200 μm. Colored images, scale bar = 100 μm. **f,** Quantification of starter PV cells (total number of rabies+ cells counted: 416, number rabies+/GFP+ starter cells: 69, and number of rabies+ only cells: 347, 2 mice). **g,** Quantification of local presynaptic cells that provided inputs to starter transplanted cells (total number of rabies+ presynaptic cells: 307, number of GABA+/rabies+ cells: 46, number of GABA-/rabies+ cells: 261, 2 mice). Rabies tracing of PV interneurons was performed in 2 PV-TVA mice.

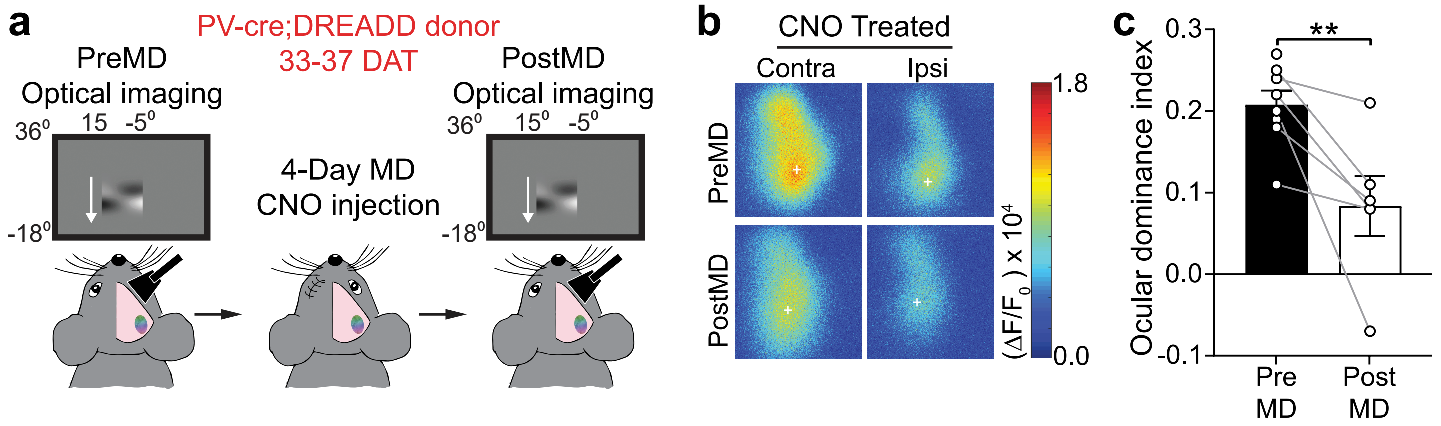

Supplementary Figure 3. Inactivation of transplanted interneurons does not prevent cortical plasticity in the recipients. a, WT adults received transplantation of MGE tissue from PV-cre;DREADD donors. Intrinsic optical imaging was performed to measure ocular dominance plasticity (ODP) in adult transplant recipients around 33-37 days after transplantation (DAT). The recipients received injections of CNO (2 dose per day at 10-12 hr interval; 4-5mg/kg) during four days of monocular deprivation (MD). **b,** Examples of response amplitude before and after 4D MD. **c,** Four-day MD resulted in a shift of ocular dominance index (ODI) in transplant recipients that were treated with CNO (PreMD: n = 8 mice, solid black; PostMD: n = 6 mice, open black; **p= 0.009, Mann-Whitney U test, two-tailed). ODI is calculated as (Contra response amplitude – Ipsi response amplitude) / (Contra response amplitude + Ipsi response amplitude). Data are mean ± SEM.

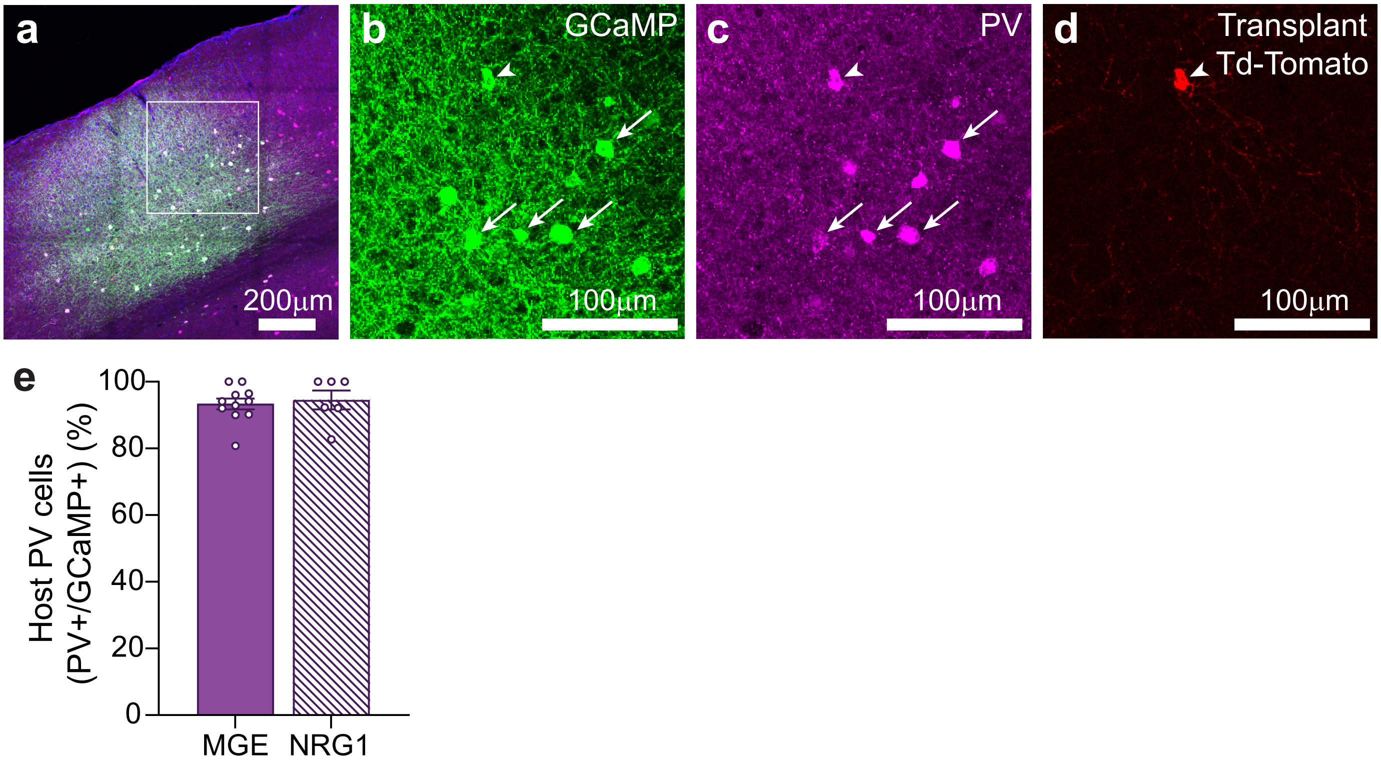

Supplementary Figure 4. Virus-labeled host cells express marker for Parvalbumin interneurons. a-d, Immunostaining of PV was performed in 5 different transplant recipients. **a,** Example coronal section from a PV-cre adult transplant recipient that received cre-dependent GCaMP6 virus injection. Scale bar = 200 µm. **b,** GCaMP6-expressing cells (green). **c,** Immunostaining for Parvalbumin confirmed that many virus-labeled host cells are PV+ (arrow; purple). **d,** Cells triple-labeled with GCaMP, PV and tdTomato were transplanted PV interneurons (arrowhead; red). Scale bars in **b**, **c**, and **d** = 100 µm. **e,** More than 90% of virus-labeled cells co-expressed PV in both MGE (n = 11 sections from 3 mice, solid purple) and NRG1 groups (n = 6 sections from 2 mice, dashed purple). Cells that also expressed tdTomato were not included in the quantification. Data are mean ± SEM.

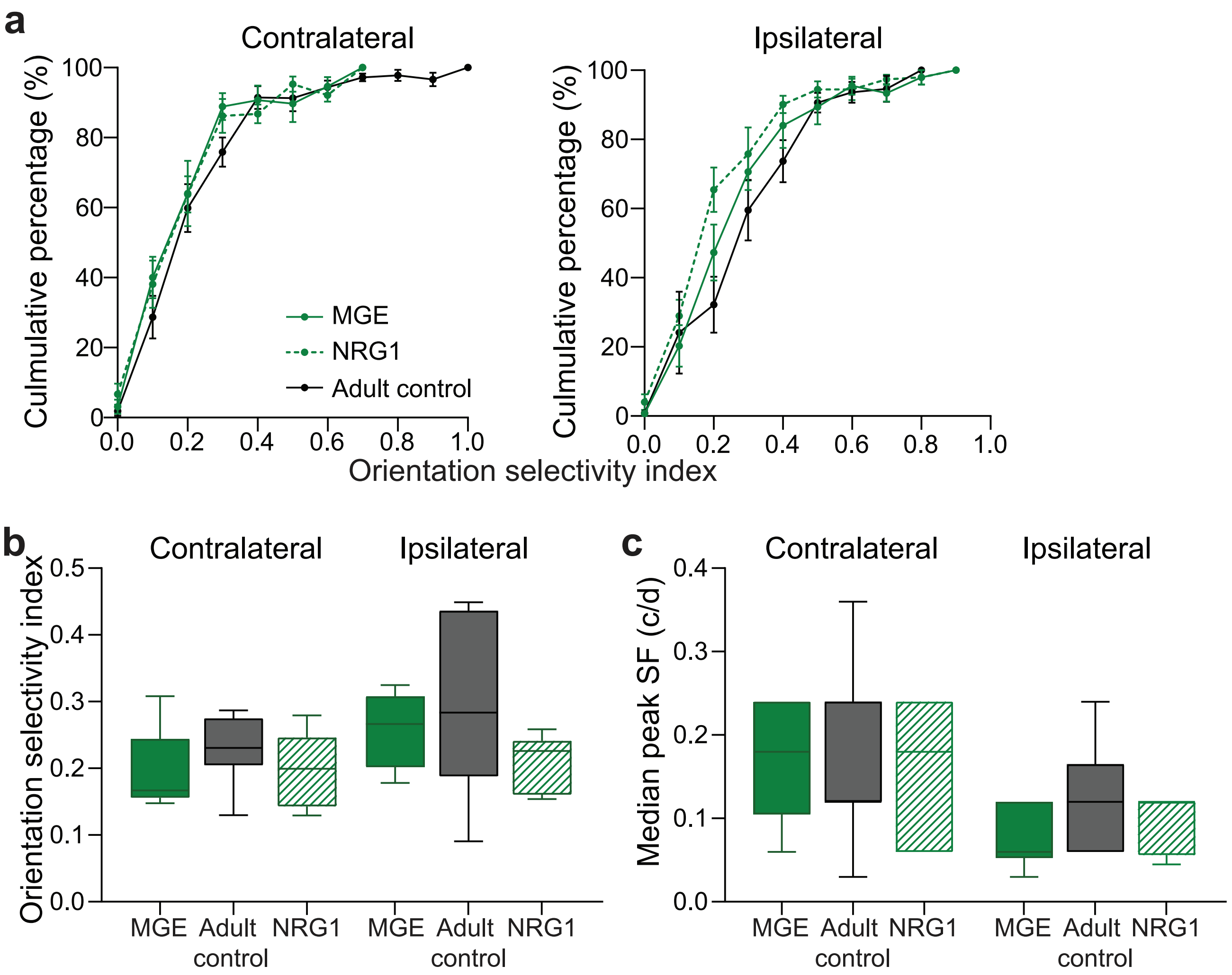

Supplementary Figure 5. Interneuron transplantation does not alter the tuning properties of host neurons. a, Left, cumulative distribution of orientation selectivity index (OSI) before monocular deprivation for contralateral responses of host PV interneurons in MGE transplant recipients (MGE, green, n = 6 mice), age-matched adult control (Adult control, black, n = 8 mice), and MGE transplant recipients treated with NRG1 (NRG1, dashed green, n = 6 mice). Right, cumulative distribution of OSI for ipsilateral responses of PV interneurons. Data are mean ± SEM. **b,** Box plots of median orientation selectivity index. Interneuron transplantation did not alter orientation tuning of adult host PV interneurons. There was also no difference in orientation tuning between MGE and NRG1 group before MD as expected, since these transplant recipients were randomly assigned to the experimental groups and NRG1 treatment started during MD (Contralateral: MGE, n = 6 mice; Adult control, n = 8 mice; NRG1, n = 6 mice; p = 0.358, Kruskal-Wallis one-way ANOVA; Ipsilateral: MGE, n = 6 mice; Adult control, n = 8 mice; NRG1, n = 6 mice; p = 0.303, Kruskal-Wallis ANOVA). **c,** Box plots of median peak spatial frequency. Interneuron transplantation did not alter spatial frequency tuning of adult host PV interneurons. (Contralateral: MGE, n = 6 mice; Adult control, n = 8 mice; NRG1, n = 6 mice; p = 0.966, Kruskal-Wallis one-way ANOVA; Ipsilateral: MGE, n = 6 mice; Adult control, 8 mice; NRG1, n = 6 mice; p = 0.317, Kruskal-Wallis ANOVA). For box plots in **b** and **c**, the bottom and top whiskers represent the minimum and maximum values, respectively, the central mark indicates the median, and the bottom and top edges of the box indicate 25th and 75th percentiles, respectively.

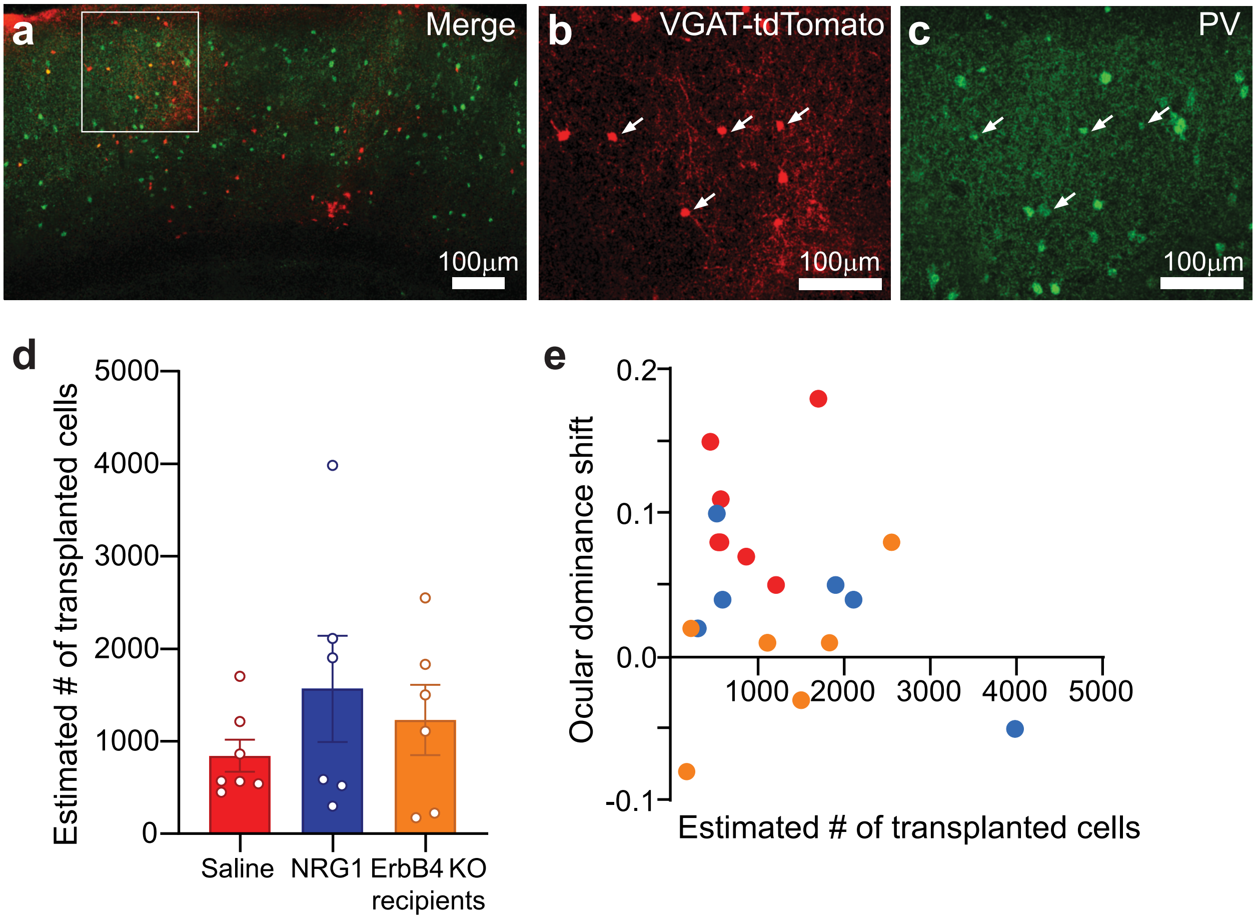

Supplementary Figure 6. The extent of ODP is not correlated with the number of transplanted cells. a, An example coronal section from an adult mouse that received VGAT-tdTomato MGE transplantation. Transplanted VGAT+ cells (red) dispersed throughout all layers, and a subset of the transplanted cells expressed marker for mature PV interneurons (green). Immunostaining of PV was performed in brain sections from 3 different VGAT-tdTomato MGE transplant recipients. **b-c,** Magnified images of the region outlined in **a**. **d,** Quantification of VGAT-tdTomato-positive transplanted cells (Saline: n = 7 mice, red; NRG1: n = 6 mice, blue; PV-cre;ErbB4$^{flx/flx}$ recipients: n = 6 mice, orange. p = 0.690, Kruskal-Wallis one-way ANOVA). The data are mean ± SEM. **e,** Estimated number of transplanted cells plotted against ocular dominance shifts for recipients injected with saline (red) or NRG1 (blue), and PV-cre;ErbB4$^{flx/flx}$ recipients (orange). There was no correlation between the number of transplanted cells and ocular dominance shift (R^2 = 0.234).

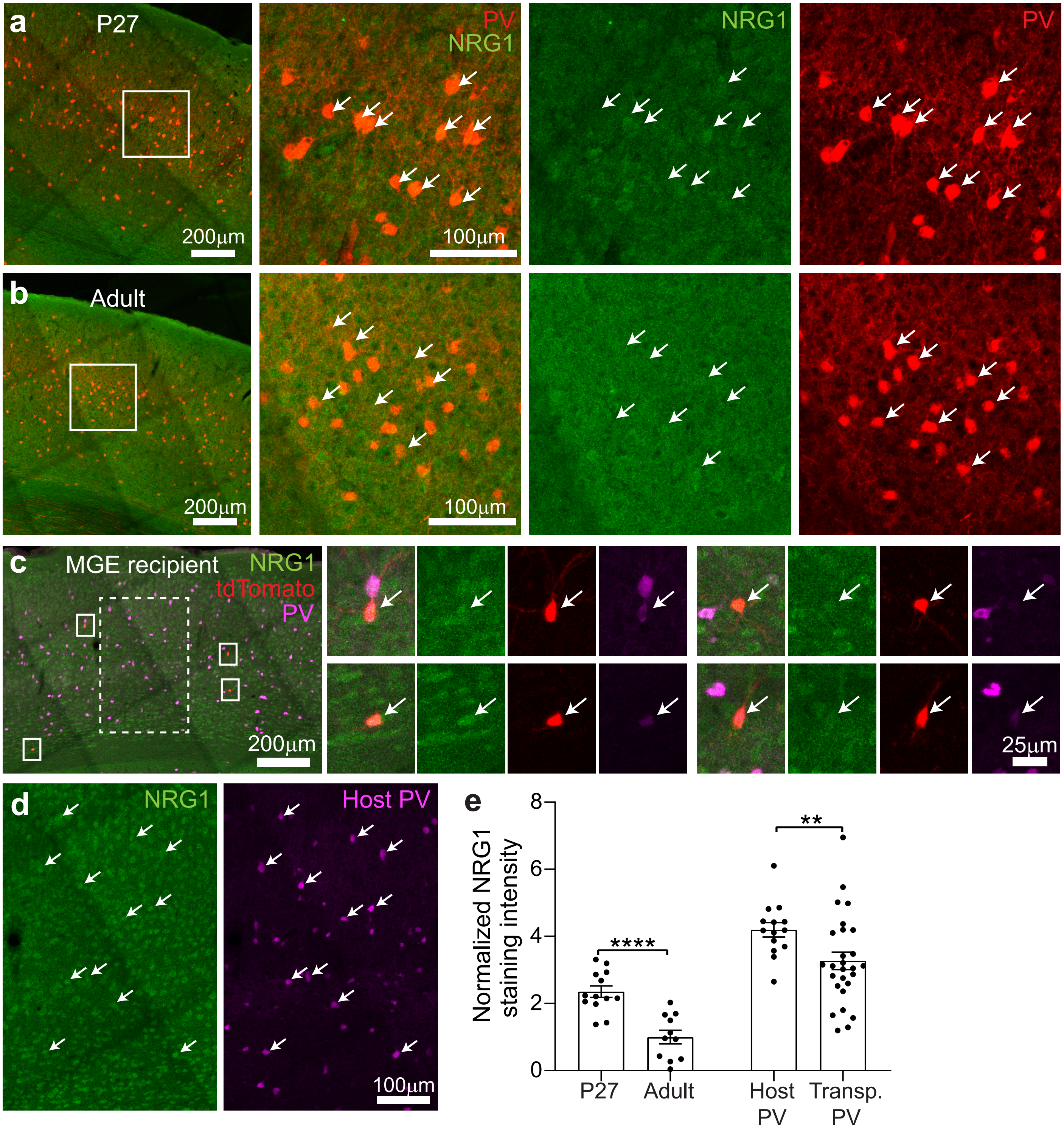
a
P27
200µm
PV
NRG1
100µm
NRG1
PV
b
Adult
200µm
100µm
c
MGE recipient
NRG1
tdTomato
PV
200µm
25µm
d
NRG1
Host PV
100µm
e
Normalized NRG1 staining intensity
8
6
4
2
0
****
**
P27
Adult
Host PV
Transp. PV

Supplementary Figure 7. Interneuron transplantation elevates the level of NRG1 within host PV interneurons

a-b, Confocal images of PV expression (red) and NRG1 immunostaining (green) in coronal sections from P28 (**a**), and adult (**b**, >P170) PV-tdTomato mice. Immunostaining was performed in 13 brain sections from 3 P28 mice and in 11 brain sections from 3 adult mice. **c,** Confocal images of PV (purple) and NRG1 (green) immunostaining and tdTomato expression. Cells that co-expressed PV and tdTomato were transplanted PV interneurons. Solid boxes indicate four example transplanted cells, and the magnified views are shown in small panels to the right. Dashed box indicates the host PV cells and the magnified view is shown in **d**. **d,** Magnified view of NRG1 immunostaining in host PV interneurons. Scale bar = 100 µm. Immunostaining of NRG1 in transplanted (**c**) and host (**d**) cells were performed in brain sections from the same 3 mice. **e,** Quantification of NRG1 expression intensity in PV interneurons in critical period (P27: n = 13 sections from 3 mice) and adult mice (n = 11 sections from 3 mice) as well as in transplant recipients at 35DAT (Host transplant: n = 14 sections from 3 mice; transplant PV: n = 28 sections from 3 mice). The mean expression intensity for each group was normalized to the mean intensity value for adult sections. In normal PV interneurons, the intensity of NRG1 expression decreases with age (P27 vs. Adult: ****p = 0.00008, Mann-Whitney U test, two-tailed). Interneuron transplantation elevates the intensity level of NRG1 in the host PV interneurons. (Host PV vs. Transplanted PV: **p = 0.009, Mann Whitney U test, two-tailed).
